# A chromosomal connectome for psychiatric and metabolic risk variants in adult dopaminergic neurons

**DOI:** 10.1186/s13073-020-0715-x

**Published:** 2020-02-19

**Authors:** Sergio Espeso-Gil, Tobias Halene, Jaroslav Bendl, Bibi Kassim, Gabriella Ben Hutta, Marina Iskhakova, Neda Shokrian, Pavan Auluck, Behnam Javidfar, Prashanth Rajarajan, Sandhya Chandrasekaran, Cyril J. Peter, Alanna Cote, Rebecca Birnbaum, Will Liao, Tyler Borrman, Jennifer Wiseman, Aaron Bell, Michael J. Bannon, Panagiotis Roussos, John F. Crary, Zhiping Weng, Stefano Marenco, Barbara Lipska, Nadejda M. Tsankova, Laura Huckins, Yan Jiang, Schahram Akbarian

**Affiliations:** 1 Department of Psychiatry, Icahn School of Medicine at Mount Sinai, New York, NY USA; 2 Friedman Brain Institute, Nash Family Department of Neuroscience, Icahn School of Medicine at Mount Sinai, New York, NY USA; 3 J.J. Peters Veterans Affairs Hospital, Bronx, NY USA; 4 Department of Genetics and Genomics, Icahn School of Medicine at Mount Sinai, New York, NY USA; 5 Pamela Sklar Division of Psychiatric Genomics, Icahn School of Medicine at Mount Sinai, New York, NY USA; 6 Human Brain Collection Core, National Institute of Mental Health, Bethesda, MD USA; 7 MDPhD Program in the Graduate School of Biomedical Sciences, Icahn School of Medicine at Mount Sinai, New York, NY USA; 8 New York Genome Center, New York, NY 10013 USA; 9 Program in Bioinformatics and Integrative Biology, University of Massachusetts Medical School, Worcester, MA 01605 USA; 10 Department of Pathology, Icahn School of Medicine at Mount Sinai, New York, NY USA; 11 Department of Pharmacology, Wayne State University, Detroit, MI USA

**Keywords:** Spatial genome, chrom3D, Euclidean hot spots, Shared nuclear territories, BMI GWAS, Schizophrenia GWAS, Dopamine, Neurons, Obesity, Schizophrenia, Metabolic syndrome

## Abstract

**Background:**

Midbrain dopaminergic neurons (MDN) represent 0.0005% of the brain’s neuronal population and mediate cognition, food intake, and metabolism. MDN are also posited to underlay the neurobiological dysfunction of schizophrenia (SCZ), a severe neuropsychiatric disorder that is characterized by psychosis as well as multifactorial medical co-morbidities, including metabolic disease, contributing to markedly increased morbidity and mortality. Paradoxically, however, the genetic risk sequences of psychosis and traits associated with metabolic disease, such as body mass, show very limited overlap.

**Methods:**

We investigated the genomic interaction of SCZ with medical conditions and traits, including body mass index (BMI), by exploring the MDN’s “spatial genome,” including chromosomal contact landscapes as a critical layer of cell type-specific epigenomic regulation. Low-input Hi-C protocols were applied to 5–10 × 10^3^ dopaminergic and other cell-specific nuclei collected by fluorescence-activated nuclei sorting from the adult human midbrain.

**Results:**

The Hi-C-reconstructed MDN spatial genome revealed 11 “Euclidean hot spots” of clustered chromatin domains harboring risk sequences for SCZ and elevated BMI. Inter- and intra-chromosomal contacts interconnecting SCZ and BMI risk sequences showed massive enrichment for brain-specific expression quantitative trait loci (eQTL), with gene ontologies, regulatory motifs and proteomic interactions related to adipogenesis and lipid regulation, dopaminergic neurogenesis and neuronal connectivity, and reward- and addiction-related pathways.

**Conclusions:**

We uncovered shared nuclear topographies of cognitive and metabolic risk variants. More broadly, our PsychENCODE sponsored Hi-C study offers a novel genomic approach for the study of psychiatric and medical co-morbidities constrained by limited overlap of their respective genetic risk architectures on the linear genome.

## Background

Midbrain dopaminergic neurons (MDN), loosely organized into three developmentally and anatomically defined clusters—substantia nigra pars compacta (SNpc/A9), ventral tegmental area (VTA/A10), and the retro-rubral field (RRF/A8) [1–3]—critically regulate normal and diseased cognition [4], together with reward-associated behaviors, and food intake and appetite-related metabolic homeostasis [5–7], among other functions. By generating a list of *cis*-regulatory sequences identified as active enhancers associated with MDN gene expression, a recent laser capture microdissection study reported significant enrichment for sequences conferring heritable liability for disorders and traits associated with mood and psychosis spectrum disorders including schizophrenia and depression, reward behaviors, and metabolism [8]. This apparent functional convergence of the genetic risk architectures of cognitive [4] and metabolic [7, 8] disorders within a specific cell type—the MDN—is of clinical relevance, given that metabolic sequelae, including excess body mass index [9], impaired glucose homeostasis [10] and dyslipidemias [11, 12] (as well as their co-occurrence, clinically termed “metabolic syndrome” [13]), significantly contribute to medical co-morbidities and early mortality, with 15–20-year gaps in life expectancy in subjects diagnosed with schizophrenia as compared to healthy controls [14–16]. However, cell type-specific cross-disorder exploration of the genomic risk architectures of schizophrenia and excess BMI and other metabolic traits is challenging [17] as these conditions show, on a genome-wide scale, only very limited or even discordant overlap based on cross-disorder correlation methods including LD score regression or correlation of polygenic risk scoring [18–20].

Given these limitations of LD core regression, polygenic risk scoring, and methodologies confined to the “linear genome” approach while not taking cell type into account, we hypothesized that mapping the MDN “spatial genome,” including chromosomal conformations that shape local chromatin environments and cell-specific gene expression programs, could provide deeper insights into genomic interactions at the site of risk variants associated with psychiatric and metabolic disease and ultimately uncover regulatory mechanisms underlying the co-morbidity of both phenotypes. Indeed, chromosomal contact mapping via DNA-DNA proximity mapping by fragmentation-religation, commonly referred to as Hi-C [21], is a powerful approach to chart loop-bound regulatory non-coding DNA in developing or adult brain [22–24], including risk sequences contributing to psychiatric and cognitive disease [24–29]. Unfortunately, however, such type of approach was until now limited to Hi-C protocols requiring a very large numbers of cells (or nuclei), in the range of 10^6^–10^7^ as input [30, 31], which allows for spatial genome mapping in tissue homogenates from large forebrain structures such prefrontal or temporal cortex [32] or the fetal ventricular/subventricular zone and cortical plate [24]. However, this is impracticable for cell type-specific Hi-C on dopaminergic neurons, because an adult human brain is estimated to harbor only 0.5–2 × 10^6^ MDN, with considerable inter-individual variabilities in absolute MDN cell counts [3, 33]. Thus, in order to map the spatial genome from rare cell types, including the 4–6 × 10^5^ MDN [34] which comprise only 0.0005% of the 8–10^9^ neurons residing in a human brain [35, 36], we newly designed a simplified Hi-C protocol based on bacterial Tn5 transposase-based [37] chromatin fragmentation applicable to as few as 5000 postmortem brain nuclei that also underwent FACS-sorting by cell type-specific nuclear markers prior to spatial genome mapping. In addition, we processed such type of material with a commercially available (Arima) Hi-C kit. We show that our low-input Hi-C protocols applied in situ (with nuclei left intact during restriction digest-fragmentation and religation) deliver chromosomal contact maps at resolutions approximating those of a conventional in situ Hi-C protocol [30] requiring 500–1000-fold higher numbers of nuclei as starting material. We then mapped, for the first time, the 3D genome of adult MDN nuclei, together with cell type-specific nuclear transcriptome (nucRNA-seq) profiling. Using these cell-specific chromosomal contact and transcriptome maps, we then anchor risk loci associated with schizophrenia and, separately, variants associated with excess body mass index, into the spatial genome, thereby uncovering numerous intra- and cross-disorder contacts in the spatial genome of the MDN.

## Methods

### Tissue and chromatin preparations

#### Human brain tissue preparation for cell type-specific profiling

Brain tissues were provided by the Icahn School of Medicine at Mount Sinai (ISMMS) brain collection (New York, NY) and the National Institute of Mental Health (NIMH) Human Brain Collection Core (Bethesda, MD). All brain tissues were dissected from banked, de-identified, frozen adult autopsy brain material of controls with no history of neurological disease and with postmortem time < 24 h. All procedures were approved the local Institutional Review Boards (ISMMS IRB#AAAJ9652-Y1M00, protocol HS#14-01007; NIH IRB General Medicine 4, protocol 17-M-N073 and 90-M-0142). The anterior cingulate cortex was obtained from the area of the frontal lobe anterior to the rostral genu of the corpus callosum. Substantia nigra pars compacta (SNpc) including bordering portions of the VTA were dissected from coronal brain slices with 1 mm margin around the distinctively dark anatomical area with heavy neuromelanin pigmentation. For the midbrain, the current protocol included immunolabeling with NURR1 (Santa Cruz Biotechnology, sc-990) in addition to the labeling with NeuN (EMD Millipore, *MAB377X*) antibodies. Briefly, to prepare for flow cytometry (nuclei extraction, NeuN immunotagging, DAPI staining) and downstream procedures (RNA extraction, nucRNA-seq), frozen never-fixed brain tissue specimens were homogenized in ice-cold lysis buffer, resulting in the destruction of the cell membranes and extraction of nuclei and other cellular organelles. Samples destined for spatial genome mapping included an additional formaldehyde-fixation step (see Additional file 1: Supplemental Methods). The homogenate was underlaid with sucrose solution and ultra-centrifuged for 1 h; the pellet (crude nuclei fraction) was resuspended and immunotagged with NeuN (pre-conjugated with Alexa 488) and NURR1 primary antibody that had been incubated with the secondary antibody (Alexa Fluor 647 fluorochrome, Thermo Fisher, A27040) for 1 h before adding it to the nuclei suspension. Nuclei were incubated with both antibodies for 2 h; DAPI (4′,6-diamidine-2′-phenylindole dihydrochloride, Sigma Aldrich, 10,236,276,001 Roche) was added during the last 10 min. The resulting nuclei suspension was processed on a FACSAria flow cytometry sorter, after setting the appropriate gates to efficiently remove debris and dividing cells and allow for a clear separation of nuclei populations based on their fluorescence signal.

#### Nurr1 validation

Formalin-fixed brain tissue was processed with a Tissue-Tek VIP processor utilizing a standard embedding protocol. Blocks were sectioned at 5 μm on a Leica RM2255 microtome. Sections were placed on charged slides and baked overnight at 70 °C. Anti-Nurr1 was produced using the epitope of the 13 amino acid antigenic peptide (c-FYLKL EDLVP PPA) derived from the ligand-binding domain (carboxyl terminal) of NR42A (NURR1); these residues are 100% identical in human, rat, and mouse. IHC against rabbit anti-NURR1 antibody was performed on a Ventana Benchmark XT utilizing an Ultraview Universal DAB detection kit. Antigen retrieval with CC1 (Tris/Borate/EDTA buffer, pH 8.0–8.5) was performed for 1 h followed by primary antibody incubation for approximately 30 min. Further validation was performed blotting 50 μg of human SNpc protein homogenate with affinity rabbit anti-NURR1 antibody and compared with midbrain protein homogenate of the surrounding SNpc region. β-actin was used as loading control (rabbit mAb, Cell Signaling # 4970S). As expected, ~ 67 kDa NURR1 protein was enriched in SNpc lysate compared to the control.

#### Mouse brain tissue preparation

C57BL/6 mice (JAX, Stock No. 000644) were sacrificed by cervical dislocation following anesthetization via isoflurane, and brains were harvested and fresh frozen at − 80 °C. For experiments, cerebral cortices were removed bilaterally by manual dissection, and nuclei were pelleted via ultracentrifugation at 24000 rpm for 1 h at 4 °C. Samples were sorted following labeling with anti-NeuN-488 antibody (EMD Millipore, MAB377), as previously described [23].

#### Hi-C nuclei sorting

Samples destined for Hi-C included a fixation step and therefore a FACS sorting protocol different from the one described above (for nucRNA-seq) was used. Fresh 37% formaldehyde (108 μL) was added to 4 ml of homogenate solution, followed by inversion and rotation at room temperature for 10 min. Then, 500 μL of 2M glycine was added, followed by another rotation for 5 min. Next, the homogenate was tabletop-centrifuged at 4000 rpm for 5 min in 4°. The pellet was then resuspended in 1 mL of lysis buffer. Following next was the addition of 4 mL of lysis buffer and 5 mL of sucrose buffer and inversion of the mixture. The mixture was then tabletop-centrifuged at 4000 rpm for 10 min at 4°. Next, the pellet was resuspended in 1000 μL of 0.1 BSA in DPBS and filtered through a 100-μm cell strainer. The nuclei were immunotagged with NeuN (pre-conjugated with Alexa 488) and NURR1 primary antibody (N4664) that had been incubated with the secondary antibody (Alexa 647) for 1 h before adding it to the nuclei suspension. Nuclei were incubated with both antibodies for 2 h and DAPI was added at 2 h mark. Next, the nuclei were filtered through 5 mL polystyrene tube with 35-μm cell-strainer cap. The resulting nuclei suspension was processed on a FACSAria flow cytometry sorter, after setting the appropriate gates to efficiently remove debris and dividing cells and allow for a clear separation of nuclei populations based on their fluorescence signal.

#### ^*Tn5*^Hi-C

A detailed step-by-step protocol and vendor information is provided in Additional file 1: Supplemental Methods. Briefly, nuclei were—after tissue extraction, fixation, immunotagging, and sorting—digested with MboI and re-ligated with T4 DNA ligase. After ligation, nuclei were treated with Tn5 transposase carrying Illumina sequencing adaptors Nextera kit at 37 °C for 30 min, followed by reverse crosslinking, RNase A, and proteinase K digestion. DNA was purified and libraries were prepared directly by PCR amplification with Index 5 and Index 7 primers from Illumina Nextera kit (FC-121-1030). ^*Tn5*^HiC libraries typically included DNA fragments ranging from 150 bp to 1200 bp length, with two peaks at 200 bp and 1000 bp, respectively. Ampure beads were used for size selection to collect two fractions of different length: 150–500 bp and 800–1200 bp. Libraries were 75 bp paired-end sequenced to generate 27–424 million read pairs for each library generated (Additional file 2: Table S1).

#### ^Arima^Hi-C

For one midbrain specimen, 6131 sorted dopaminergic neuronal nuclei (NeuN^+^/Nurr1^+^) and 50,000 sorted glia (NeuN^−^/Nurr1^−^) were processed using the Arima-HiC Kit User Guide for Mammalian Cell Lines (A51008) (San Diego, CA) according to the manufacturer’s instructions. They were subsequently purified using Beckman Coulter AMPure® SPRIselect Beads (Indianapolis, IN) according to the manufacturer’s instruction. Next, samples were sonicated using Covaris S220 (Woburn, MA) to a target of 300–500 base pairs. They were subsequently purified again for size selection using Beckman Coulter AMPure® SPRIselect Beads (Indianapolis, IN) according to the manufacturer’s instruction to a target of 300–500 base pairs. The DNA was then enriched for biotin using the Arima-HiC Kit Library Preparation using Swift Biosciences® Accel-NGS® 2S Plus DNA Library Kit (San Diego, CA) according to the manufacturer’s instructions. Next, the Swift Biosciences Accel-NGS 2S Plus DNA library kit (21024) (Ann Arbor, MI) was used for end repair and adapter ligation according to the manufacturer’s instructions. A unique index from the Swift Biosciences 2S Indexing Kit (26148) was ligated to each sample. DNA libraries were amplified using Kapa Hyper Prep Kit (NC0709851) (Wilmington, MA) and purified using Beckman Coulter AMPure® SPRIselect Beads according to the manufacturer’s instructions.

#### “Conventional” Hi-C

An average of 1–3 million nuclei were fixed and extracted from mouse cerebral cortex and human postmortem anterior cingulate cortex and sorted into NeuN^+^ (neuronal) and NeuN^−^ (non-neuronal) populations, which were then processed using the in situ Hi-C protocol [30], with minor modifications. Briefly, the protocol involves a restriction digest of the cross-linked chromatin within intact nuclei, followed by biotinylation of the strand ends, re-ligation, sonication, and size selection for 300–500 bp fragments, followed by standard library preparation for Illumina 125 bp paired-end sequencing, at 121–350 million paired read depth (Additional file 2: Table S1).

#### Nuclear transcriptome profiling (nucRNA-seq)

Never-fixed nuclei were FACS sorted directly into Trizol LS reagent (ThermoFisher, 10296028), and the final volume was adjusted with in 1× PBS at the volume ratio of 3 Trizol LS to 1 nuclei/PBS solution. Nuclei lysate was then mixed with an equal volume of 100% ethanol and loaded to Zymo-Spin IC Column from Direct-zol RNA MicroPrep kit (Zymo Research, R2060), and RNA is extracted following the manufacturer’s instructions. DNase I treatment was done in-column for 15 min at room temperature to remove genomic DNA. The quantity and quality of nuclei RNA were checked on Bioanalyzer using Agilent RNA 6000 Pico Kit. Nuclei RNA-seq libraries were generated by using SMARTer Stranded RNA-Seq kit following manufacturer’s instructions (Clontech, #634836). In brief, RNA is fragmented and denatured at 94 °C for 3 min followed by first-strand cDNA synthesis. The 3′ end of newly synthesized single strand cDNA will be labeled with a short nucleotide stretch introduced by the SMARTer Standed Oligo and cDNA then amplified by 12 cycles of PCR using Illumina indexing primer set. The final RNA-seq library (ribosomal depleted) was then purified by SPRI AMPure beads at 1:1 ratio to remove primer dimer (~ 83 bp), and the average size of libraries is ~ 300 bp. Human cell type-specific nuclear RNA-seq libraries were processed as paired-end read for sequencing (Additional file 2: Table S2).

### Data analysis

#### RNA-seq

Paired-end samples were considered as single-end and were mapped to human (GRCh38.p10_v26) with STAR (v2.5.3a) using a two-method step protocol following tool specifications [38]. Samples were counted by exon using featureCounts (subread v.1.5.2). RNA-SeQC [39] analyses, for quality control, confirmed much higher proportions of intra-genic (versus intergenic) reads in our nuclear RNA-seq (nucRNA-seq) datasets as expected, generated from the FACS-sorted nuclei (Additional file 3: Figure S1).

Resulting counts table was provided to the edgeR wrapper tool RUVseq [40] for differential analysis. Dopaminergic enriched genes could be observed in the differential analysis comparing Nurr1^+^/NeuN^+^ versus cortical anterior cingulate NeuN^+^, cortical anterior cingulate NeuN^−^, midbrain Nurr1^−^/NeuN^+^, midbrain Nurr1^+^/NeuN^−^, and midbrain Nurr1^−^/NeuN^−^ (Additional file 2: Table S2). No batch correction was required, except for the comparisons of midbrain Nurr1^−^/NeuN^+^ and Nurr1^+^/NeuN^−^, using RUVr and RUVg strategies. Cell specificity was assessed by a cluster analysis by PCA of 45 dopaminergic curated genes [41] over all cell populations, observing a clear clustering of the different cell types here studied. Furthermore, cell specificity was estimated using Neuroexpresso single-cell brain RNA-seq curated database [42] using *makerGeneProfile* utility (https://github.com/PavlidisLab/markerGeneProfile).

#### Transcriptomic GWAS association

Multimarker Analysis of GenoMic Annotation (MAGMA) [43], version 1.06b, was used to quantify gene expressed enrichment of Nurr1^+^/NeuN^+^ and Nurr1^−^/NeuN^−^ for a variety of GWAS traits [44–51]. For each gene and trait, MAGMA calculates the joint association of all SNPs to the gene region while it accounts for linkage disequilibrium (LD) between SNPs. The gene regions were defined with the window size of 35 kb upstream and 10 kb downstream, and LD was estimated from the European panel of 1000 Genome Project phase 3 [52]. These associations in the form of aggregated *p* values are then used for gene-set analysis, with Benjamini-Hochberg to control for multiple comparisons.

#### HiC mapping, filtering, and normalization

All libraries were mapped to either human (GRCh38.p10_v26) or mouse (GRCm38p5_M13) assemblies, filtered, and ICED normalized using the HiC-Pro tool [53] (v2.9.0). Library QC measures are reported in Additional file 2: Table S1. Minor modifications included the following: for ^*Tn5*^HiC libraries, the ligation site was set as “GATC” as blunt-ending was not performed. For HiC blunt-ended libraries using mboI, the corresponding ligation site was “GATCGATC.” For HiC libraries using Arima Kit protocol, the corresponding ligation site was as follows: “GAATAATC, GAATACTC, GAATAGTC, GAATATTC, GAATGATC, GACTAATC, GACTACTC, GACTAGTC, GACTATTC, GACTGATC, GAGTAATC, GAGTACTC, GAGTAGTC, GAGTATTC, GAGTGATC, GATCAATC, GATCACTC, GATCAGTC, GATCATTC, GATCGATC, GATTAATC, GATTACTC, GATTAGTC, GATTATTC, GATTGATC.” In order to compare Tn5-HiC with HiC, libraries were subsampled and bootstrapped using Fastq_bootstrapper utility (https://github.com/sespesogil/Fastq_bootstrapper).

#### Topological associated domains (TADs) and A/B compartment comparison

In order to compare the number and average size of TADs between different techniques, HiC libraries were subsampled to the same number of ^*Tn5*^HiC reads in order to avoid any possible bias of read coverage in the analysis. Only autosomal chromosomes were considered. TADtree (http://compbio.cs.brown.edu/projects/tadtree/) was used with predefined parameters for both libraries and species: *S* = 50, *M* = 25, *p* = 3, *q* = 13, gamma = 500, and *N* = 400. A/B compartments were called using Eigenvector utility [54], and Loess regression was performed in other to compare both techniques.

#### ^*Tn5*^-HiC and HiC interaction matrix heatmap and arc visualization

Heatmap interaction matrices were plotted using Juicer tools, and loop arc interactions were produced using HiCpro-WashU utility (https://github.com/sespesogil/HiCpro_WashU) to produce pairwise interaction tracks to be visualized in the Epigenome WashU browser (http://epigenomegateway.wustl.edu/browser/).

#### In silico 3D conformation using chrom3D

Required dopaminergic gtrack file to run chrom3D [55] was produced using the chrom3D wrapper automat_chrom3D utility (https://github.com/sespesogil/automat_chrom3D). Chromosome Y was excluded as the number of beads was not sufficient to run the model. Domains were called using Arrowhead (Juicer tools 1.7.6 [54]). “--ignore_sparsity” parameter was used, and calls could be only produced at not lower than 50 kb. At that resolution, 3066 domains were called with an average size of 1.3 Mb. A benchmark analysis was performed to determine the best number of iterations to be used. Stabilization of the model was found after 1M of iterations shown by the loss-score calculation, with greater confidence around 4M of iterations (Additional file 3: Figure S2). For the present study, we finally selected 5M iterations including the parameter “--nucleus” to force the beads to remain confined inside the designed radius: “-r 3.0”. Domain coloring was produced by automat_color (https://github.com/sespesogil/automat_chrom3D_colors) that allows to color any region of interest in the model. The gtrack model and current model are available in Additional file 2: Table S3.

#### BMI + SCZ domains

Significant BMI SNPs [48] (289 hg18 index SNPs lifted-over resulting in 289 hg38 index SNPs) and schizophrenia risk loci [56] (hg18 145 risk loci lifted-over resulting in 139 hg38 risk loci) were here used to study their spatial conformation in the 3D model (Additional file 2: Table S4). Both of the studies were built from the same population ethnicity, with the exception of a small subset of BMI SNPs corresponding to all ancestries (~ 12%; 37/257 SNPs). The average distance between schizophrenia loci (average = ~ 15.6 Mb ± 16.7, min = 268 kb, max = 85 Mb) and between BMI SNPs (average = ~ 8.5 Mb ± 9.2, min = 501 kb, max = 74.1 Mb) further confirms that the vast majority of risk sequences in each condition do not fall into the same block. Therefore, the majority risk/trait loci are independent. Of note, GWAS studies generally do not report haplotype association, and haplotype seems to be disconnected to gene regulatory mechanisms and chromatin interactions [57, 58]. Furthermore, each BMI and schizophrenia risk locus that fell into a chromatin domain that harbors both schizophrenia and BMI risk sequences was limited most of the time to a single domain (Additional file 3: Figure S3). Thus, the present study focused in the encapsulation of both traits into topological associated domains as “blocks” generally sharing mechanisms of co-regulation [59]. Of note, the majority of domains co-localizing BMI and schizophrenia traits across cell types are conserved (Additional file 3: Figure S3); hence, we do not expect that this co-localization is particularly specific for Nurr1^+^/NeuN^+^, as opposed to the cell-specific regulation of the spatial configuration of domains inside the cell nucleus. Each feature was intersected (bedtools/2.24.0) with the haploid version of the 3D model finding 53 haploid domains common for both traits, described in the present study as Euclidean hot spots or “EH.” However, the diploid model could only harbor 100 domains of them, as 6 of them were discarded from chrom3D run. Euclidean hot spots were defined by hierarchical clustering using R package “pheatmap.” To estimate the reliability of these hot spots and spatial conformation, multiple runs of chrom3D iterations were produced (12 runs, from 250k, 500k, 1M to 10M of iterations).

#### Random shuffling

The null hypotheses of finding EHs with the same pairwise distances between associated domains was tested against randomness inside the common BMI + SCZ 100 domains space, selecting random domains of the same size of the EH to be tested using the R function “*sample*.” As these pairwise distances were not following a normal distribution, tested by a Shapiro and Andersen analysis, the reliability significance of finding assessed Euclidean distances inside each EH was determined by a Wilcoxon test (Additional file 3: Figure S2). Furthermore, to determine the specificity of the Euclidean space to BMI and schizophrenia, corresponding haploid hot spot versions were intersected with other significant GWAS polymorphisms features/disorders/diseases (Additional file 3: Figure S4).

#### Domain Euclidean distances

Euclidean pairwise “*straight-line*” distances among beads (domains) carrying both BMI and SCZ risk variants and domain distance to the centroid were calculated using the utility automat_euclidean (https://github.com/sespesogil/automat_euclidean) that allows to calculate any Euclidean pairwise distance calculation from any region of interest in the model. Cross comparison among cell types was performed considering both alleles separately and mapping ^*Tn5*^HiC Nurr1^+^/NeuN^+^ hot spots to the chrom3D in silico models generated from Hi-C datasets for nine other cell types and from the ^Arima^HiC dataset generated from midbrain Nurr1^+^/NeuN^+^. Distances among domains were considered only if they belong to different chromosomes to do not overestimate the distances among continuous domains, as the number of domains regarding the same genomic region depends on each cell type (some datasets might have several domains per each ^*Tn5*^HiC domain called).

#### Risk loci interactome

Circos plots showing disease-relevant interactions at 40 kb within and across domain were produced using the tool risk loci_interactome utility (https://github.com/sespesogil/risk_loci_interactome). Normalized frequencies from HiC-Pro forward and reverse interactions were called by risk variants (Additional file 2: Table S5). To identify significantly enriched interactions involving a bin of interest with another bin, our principal approach was to first estimate the expected interaction counts for each interaction distance by calculating the mean of all intra-chromosomal bin-bin interactions of the same separation distance throughout the raw intra-chromosomal contact matrix. We used the R package, HiTC [60], to facilitate manipulation of our HiC-Pro-produced raw contact matrices and estimation of the expected counts at various interaction distances. The probability of observing an interaction between a bin-of-interest and another bin was then defined as the expected interaction between those two bins divided by the sum of all expected interactions between the bin-of-interest and all other intra-chromosomal bins. A *p* value was then calculated as binomial probability of observing the number of interaction counts or more between the bin-of-interest and some other bin where the number of successes was defined as the observed interaction count, the number of tries as the total number of observed interactions between the bin-of-interest and all other intra-chromosomal bins, and the success probability as the probability of observing the bin-bin interaction estimated from the expected mean interaction counts. The Benjamini-Hochberg method was used to control false discovery rate (FDR) for *p* values determined for all interactions with a bin-of-interest (includes all bins 1 Mb up and downstream in our tests).

#### Chromatin loop GWAS association

To investigate if chromatin loops played a role in various diseases and traits, loop regions were tested to calculate common trait-associated genetic variants enrichment using a set of selected GWAS studies. To do so, LD score-partitioned heritability [61] was used to calculate if common genetic variants in genomic regions of interest explain more of the heritability than variants not in the regions of interest adjusting for the number of variants in either category. The approach allows for a correction of the general genetic context of the genetic regions of interest by using a baseline model of general genomic annotation (such as conserved regions and coding regions) and hence makes it possible to assess the enrichment above what is expected from the general genetic context of the genomic regions of interest. We extended those genomic regions of interests, i.e., loop regions, by 1000 base pairs on both sides to capture adjacent genetic variants and filtered out those with FDR *p* value < 0.05. The broad MHC-region (chr6, 25–35 Mb) was also removed due to its extensive LD structure, but otherwise default parameters were used for the algorithm.

#### TNE and motif analysis

We used the datasets of dopaminergic neuron transcribed non-coding elements (TNS) from laser-captured substantia nigra cells [8]. TNE expression within each EH was determined by the clamped accumulation of these elements in each in silico domain. As the majority of them fall into enhancer regions, we used these elements as proxy to determine possible mechanism of co-regulation among BMI and SCZ risk polymorphisms. Hence, we intersected TNE coordinates with chromosomal contacts interconnecting BMI and SCZ risk sequences. The resulting set of TNEs was used to run a motif analysis using Homer (v4.10) discovering both known and de novo motifs (Additional file 2: Table S6).

#### Permutation analysis of *cis*-eQTLs

Brain *cis*-expression quantitative trait loci were extracted from [62]. Associations with the disease-relevant chromosomal connectome were examined using regioneR package [62]. The likelihood of this association was estimated by the overlapping relationship among significant eQTLs (FDR < 10^−8^) using 480,499 out of 643,032 eQTL unique coordinates, and interaction bins were randomized over 10,000 permutations. To assess tissue specificity of these BMI-SCZ chromosomal interactions, GTEx eQTLs were downloaded and significant eQTLs were extracted (*q* < 0.05). Unique eQTLs across all GTEx tissues available were extracted (https://github.com/sespesogil/cross_intersection), and association was estimated following previous permutation analysis. Many eQTL association results may not be independent but instead result from high linkage disequilibrium between eQTL SNPs. To address this, clumping was performed for each gene using the PLINK 1.90 software at a clump distance of 250 kb and an *r*^2^ of 0.2 (Additional file 4: Datafile QTL analysis 1) and 0.6 (Additional file 5: Datafile QTL analysis 2). The clumped summary statistics were then tested for enrichment within the 11 Euclidean hot spots.

#### Gene ontology analysis and protein-protein interaction network

Selected gene ontology terms were produced using the Cytoscape tool ClueGO with a *p* value threshold of 0.05, Bonferroni adjusted [63] (Additional file 2: Table S7). In order to find the highest confident protein-protein interaction network, all SNP/risk loci-associated genes and transcription factors were run with a high confidence value of 0.9 and 0.95 respectively.

## Results

### Sorting and separation of midbrain cell types to enrich for MDN nuclei

**Fig. 1:**
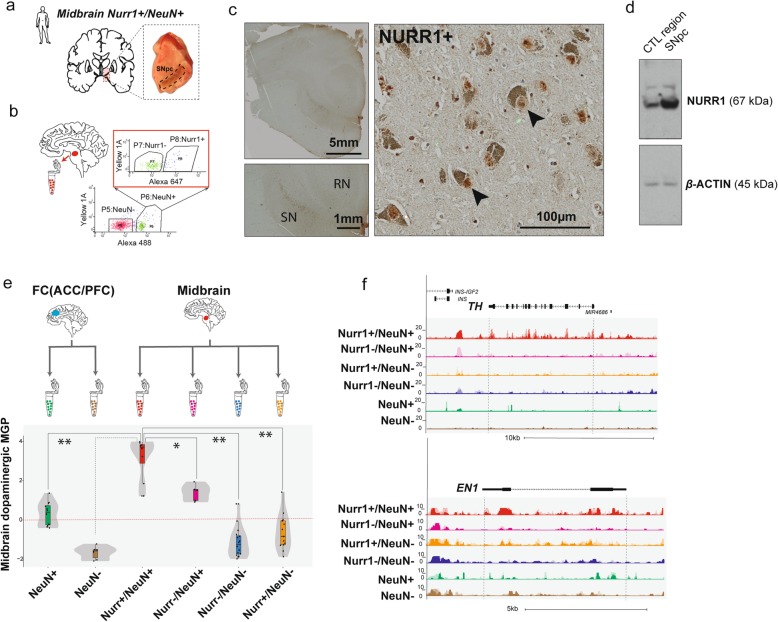
Phenotypic characterization of Nurr1^+^/NeuN^+^ dopaminergic neurons. **a** (top) Coronal midbrain section including substantia nigra (SN). **b** Representative FACS plot showing subtypes of sorted nuclei including double positive Nurr1^+^/NeuN^+^ (fraction P8). **c** Midbrain section immunohistochemically stained with anti-Nurr1 antibody, showing Nurr1 immuno-reactive nuclei associated with melanin-positive somata (black arrows) in the SN. **d** Nurr1 western blot comparing SN versus non-specific midbrain control region. **e** Dopaminergic marker genes (adopted from single-cell RNA-seq study [42]) were quantified for expression in the six cell type-specific nuclei fractions collected by FACS from ventral midbrain and anterior cingulate cortex, as indicated. Note significantly higher expression of dopaminergic marker genes (*y*-axis) in midbrain Nurr1^+^/NeuN^+^ nuclei as compared to other nuclei populations (Wilcoxon test, *p* < 5 × 10^−2^ to 10^−5^). Note the subtle increase in expression of dopaminergic marker genes in midbrain Nurr1^−^/NeuN^+^ nuclei compared to the remaining four cell types, suggesting that this fraction of nuclei represents a more heterogenous admixture of cell types including subset of dopaminergic intermingled with non-dopaminergic neuron nuclei. **f** Representative genome browser screenshots of nucRNA-seq coverage for dopaminergic marker genes, (top) *TYROSINE HYDROXYLASE* (*TH*) and (bottom) *ENGRAILED1* (*EN1*) in four midbrain nuclei fractions based on Nurr1 and NeuN immunotagging as indicated and two cortical nuclei fractions based on NeuN immunotagging as indicated: midbrain: (red track) Nurr1^+^/NeuN^+^, (pink track) Nurr1^−^/NeuN^+^; (orange track) Nurr1^−^/NeuN^+^, (blue track) Nurr1^−^/NeuN^−^; anterior cingulate cortex (ACC): green track NeuN^+^ and brown track NeuN^−^

To explore genome organization and function in adult dopaminergic neurons residing in ventral midbrain together with various other neuronal and glial subpopulations, we first designed an enrichment procedure for MDN nuclei from coronal blocks harboring SNpc/A9 and the bordering VTA/A10 area (Fig. 1a). Intact nuclei extracted from tissue were purified, double-stained, and sorted by FANS, or fluorescence-activated nuclei sorting [64, 65], with NeuN as a pan-neuronal marker and with NURR1 *nuclear receptor subfamily 4 group A member 2* (*NR4A2*), a gene essential for MDN development and maintenance [66] (Fig. 1b). Consistent with previous studies [67, 68], there was robust NURR1 immunoreactivity in the ventral midbrain, including neuronal nuclei residing in neuromelanin-rich (dopaminergic) somata (Fig. 1c, d), providing a strong rationale for using NURR1 as a cell (type)-specific immunotag. Next, we profiled the nuclear transcriptome in *N* = 30 nucRNA-seq datasets (Fig. 1e, f, Additional file 2: Table S2), including *N* = 4 Nurr1^+^/NeuN^+^ midbrain samples (5–20 × 10^3^ sorted nuclei/sample), in comparison to *N* = 6 Nurr1^+^/NeuN^−^, N = 3 Nurr1^−^/NeuN^+^, and *N* = 8 Nurr1^−^/NeuN^−^ midbrain control samples. For additional comparison, *N* = 6 NeuN^+^ and *N* = 3 NeuN^−^ samples sorted from the anterior cingulate cortex were also included, resulting in altogether six different cell types for analyses. Principal component analyses (PCA) using the genome-wide transcriptome, or a subset of 45 dopaminergic curated genes [41], revealed clustering of the six different cell types (Additional file 3: Figure S1). Importantly, the Nurr1^+^/NeuN^+^ MDN (dopamine neuron-enriched) samples showed, in comparison to each of five remaining neuronal (non-dopaminergic) and non-neuronal cell types from the midbrain and cingulate cortex, significantly higher expression of dopamine neuron-specific marker gene sets curated from pooled and single-cell transcriptome datasets for 36 major cell types of the mammalian brain [42] (Fig. 1e, f). This effect was highly specific because the Nurr1^+^/NeuN^+^ MDN did not show, in comparison to their surrounding midbrain cell populations or to the anterior cingulate cortex cells, higher expression for marker gene sets for serotonergic and cholinergic neurons or (glutamatergic) pyramidal neurons or various types of glia (Additional file 3: Figure S5). We conclude that the transcriptome of FACS-sorted midbrain nuclei fraction defined as Nurr1^+^/NeuN^+^ indeed specifically represents a dopaminergic phenotype.

### Schizophrenia and body mass index risk variants rank at the top in enrichment analyses of MDN transcriptomes but show limited overlap on the linear genome

Having confirmed that Nurr1^+^/NeuN^+^ midbrain nuclei are representative for MDN, we next compared midbrain Nurr1^+^/NeuN^+^, Nurr1^−^/NeuN^+^, Nurr1^+^/NeuN^−^, and Nurr1^−^/NeuN^−^ and cortical NeuN^+^ and NeuN^−^ transcriptomes for enrichment in genetic variants mapped in genome-wide association studies (GWAS) to 31 medical and psychiatric disorders and traits, by applying Multimarker Analysis of GenoMic Annotation (MAGMA) [43] as a gene set analysis method to perform gene set analysis based on cell-specific transcript enrichment and GWAS data as input [43, 61] (Additional file 2: Table S8; Additional file 3: Figure S6). Of note, consistent with similar observations in a broad variety of other neuronal cell types residing in the fore-, mid-, and hindbrain [69], all three neuronal subpopulations in our study, including the Nurr1^+^ and Nurr1^−^ NeuN^+^ midbrain neurons and ACC NeuN^+^ cortical neurons, showed significant enrichment for variants associated with various cognitive and metabolic traits. Thus, the transcriptome of dopaminergic neurons as defined by the midbrain’s Nurr1^+^/NeuN^+^ fraction ranked top for BMI (Additional file 3: Figure S6) and second-to-top for SCZ enrichment. The ACC NeuN^+^ ranked top for SCZ (Additional file 3: Figure S6). These disease-specific enrichments in neurons, including the MDN transcriptomes, and the a priori functional importance of the MDN both for psychosis and body weight, feeding, and metabolism [5, 6], would provide strong rationale to explore the genomic risk architectures of SCZ and BMI in cell-specific manner. However, as mentioned above, charting sequences carrying risk variants for schizophrenia and BMI on the linear genome is largely non-informative from the perspective of cross-disorder comparison [18, 19], with very few single nucleotide polymorphisms (SNPs) implicated in both conditions [70]. To re-examine and further confirm this observation, we surveyed schizophrenia GWAS summary statistics involving 105,318 subjects combined from the Psychiatric Genomics Consortium and CLOZUK [56] and counted 12/139 (< 5%) SCZ risk loci harboring one or more of the 289 risk SNPs for the 339,224 subjects BMI GWAS [48], with > 80% of SCZ risk loci separated by > 1 Mb linear genome sequence from the nearest BMI index SNP (Additional file 2: Table S4; Additional file 3: Figure S3).

### Chromosomal conformation mapping in dopaminergic neurons to explore schizophrenia and body mass index risk variants

Next, we examined the “spatial” (“3-dimensional”) genome, including territorial and intra-nuclear positioning of the chromosomal material, and its modular organization into chromatin domains extending across the 10^4^–10^7^ bp range, representing (semi-)autonomous regulatory structures constraining promoter-enhancer interactions and other transcriptional mechanisms [71, 72]. Given that both BMI and SCZ genetic risk variants show enrichment in the MDN transcriptome, such types of regulatory mechanisms governing transcription via chromosomal conformations could converge on BMI and SCZ risk sequences. Therefore, we speculated that the limited overlap between BMI and SCZ risk sequences on the “linear genome” (Additional file 3: Figure S3) does not preclude extensive and cell type-specific interactions between the genetic risk architectures of these two conditions in the spatially organized genome. To examine this, we decided to map chromosomal contacts in the MDN on a genome-wide scale using Hi-C. However, to date, chromosomal conformations in the human brain have been mapped in forebrain tissue homogenates with 10^6^–10^7^ nuclei as input for DNA-DNA proximity (Hi-C) assays [24]. However, tissue homogenate-based Hi-C would be less ideal for adult ventral midbrain, with the MDN intermingled with various other cell types and even normal aging associated with major shifts in cell type composition due to a decline in MDN numbers and glial proliferation [3, 73]. We therefore employed a newly designed Hi-C protocol applicable to as little as 5 × 10^3^ formaldehyde-fixed, immunotagged, and FANS-sorted brain nuclei. Our ^*Tn5*^Hi-C protocol, in contrast to conventional Hi-C, does not require DNA blunting, end repair, biotin incorporation, dA tailing, or sonication/shearing. Instead, the intact nuclei are sequentially exposed to restriction digest, relegation, and then Tn5 transposase treatment for single-step fragmentation of genomic DNA and concomitant attachment of sequencing adaptors [74]. After fragment size selection, the 800–1200 bp fraction of the library carried the largest fraction of chimeric reads (Additional file 3: Figure S7). Comparison of ^*Tn5*^Hi-C and standard Hi-C libraries from NeuN^+^ sorted nuclei from human postmortem, and mouse cerebral cortex (Additional file 2: Table S1) showed minor differences in genome-wide numbers and average length of self-folding (topologically associated) chromatin domains (TADs) and TAD profiles (Additional file 3: Figure S8, S9). We conclude that ^*Tn5*^Hi-C, while requiring a 1000-fold fewer nuclei from postmortem brain tissue as starting material, delivers chromatin domain maps similar to those constructed from standard Hi-C libraries. As a final test, we compared three MDN (midbrain Nurr1^+^/NeuN^+^) Hi-C libraries from three brains, two generated with our ^*Tn5*^Hi-C protocol and one with a commercial (Arima) Hi-C kit adapted for limited amounts of input, or less than 10,000 nuclei starting material (Additional file 2: Table S1, Additional file 3: Figure S10). The library-to-library Pearson correlation coefficient for genome-wide valid interaction pairs (chimeric “non-linear” reads) between the two ^*Tn5*^Hi-C libraries was 0.86, and for the ^Arima^Hi-C to ^*Tn5*^Hi-C comparisons, it was 0.80 and 0.89 (Additional file 3: Figure S10). These findings, taken together, strongly suggest our Hi-C chromosomal contact mappings built from limited amounts of starting material extracted from postmortem brain tissue provide a 3DG similar to those built with other established Hi-C protocols.

**Fig. 2:**
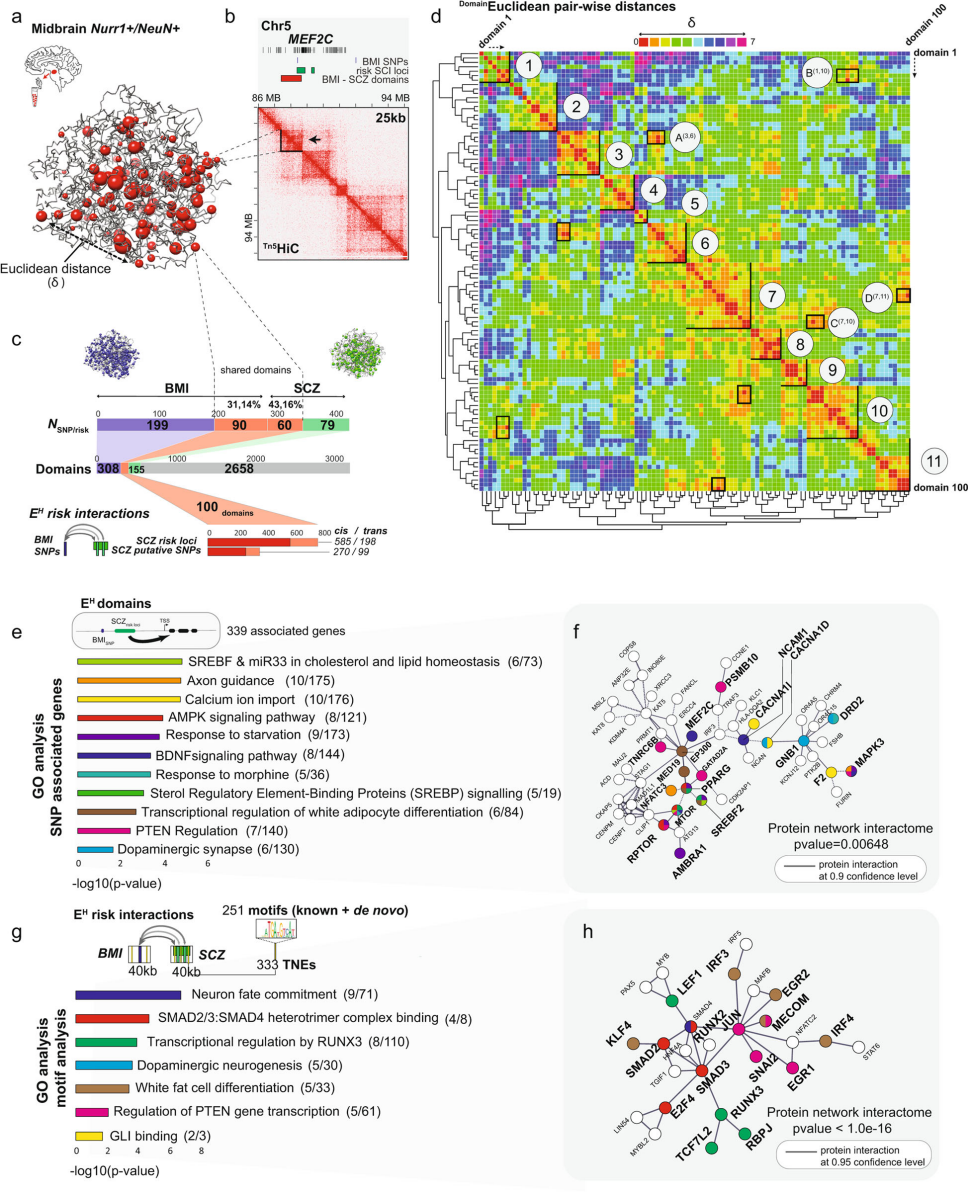
Schizophrenia and body mass index risk architectures mapped onto the spatial genome of midbrain dopaminergic neurons. **a** Nurr1^+^/NeuN^+ Tn5^HiC library (761M reads) chrom3D in silico modeling. Shared domains (red beads) harbor both BMI and SCZ risk variants. **b** Interaction matrices at 25 kb resolution, showing sharp boundaries between domains including their nested subdomains. **c** (top to bottom) BMI and SCZ summary bar plots, including as indicated, N SNPs/loci in the domains in addition to proportion of SCZ, BMI, and shared (SCZ + BMI) domains in the spatial genome model with approximately 3000 domains. **d** Euclidean pairwise distances between 100 domains with shared BMI and SCZ risk variants, defining 11 Euclidean hot spots (*EHs*) of domains that are spatially close together. δ is defined as pairwise distances of BMI + SCZ risk domains measured in Euclidean geometrical units. **e** GO analysis and selected terms of SNP-associated genes in the EHs (*N* = 339 total genes, *p* value< 0.05, Bonferroni adjusted). **f** EH gene-associated BMI-SCZ risk contacts, with String-db proteome interactome [75] of high confidence interactions (0.9), colors represent gene ontology as in Fig. 2f. **g** Significant selected GO analysis terms (*p* value< 0.05, Bonferroni adjusted) of transcription factors binding to non-coding regulatory elements (*TNE*) regions found to anchored at EH risk interactions (333 total TNEs found harboring 251 motifs). **h** EH transcription factor interactome [75] bound to TNE regions in EH risk interactions at high confidence level (0.95), colors represent the transcription factors found in the gene ontology analysis (left)

We then generated a ^*Tn5*^HiC chromosomal contact map for MDN from a merged dataset of two independent ^*Tn5*^HiC libraries with 424 and 337M 75 bp paired-end reads respectively, generated from 6000 and 7700 NeuN^+^/Nurr1^+^ FACS-sorted nuclei of two adult midbrain specimens, with additional ^*Tn5*^Hi-C maps generated for non-neuronal midbrain nuclei (Additional file 2: Table S1). Interaction matrices at 25 kb resolution showed sharply configured chromatin domain landscapes of the MDN, comprised by TADs and nested subTADs (Fig. 2a, b). Furthermore, differential interaction analyses (HICCUPS [54]) in Tn5 and Arima HiC libraries prepared for MDN NeuN^+^/Nurr1^+^, but not their surrounding non-neuronal NeuN^−^/Nurr1^−^ nuclei, revealed many cell type-specific chromosomal contacts at the site of MDN marker genes including the transcription factor and shared BMI and SCZ risk gene, *MEF2C* (Fig. 2a, b; Additional file 2: Table S9; Additional file 3: Figure S11). Importantly, enrichment analysis for such types of chromosomal contacts revealed significant enrichment for BMI and moderate enrichment for SCZ risk sequences and various other psychiatric and cognitive traits in the NeuN+/Nurr1+ MDN but not their surrounding non-neurons (Additional file 2: Table S9; Additional file 3: Figure S11). As a third method, we applied locus-specific binomial statistics-based comparisons of chromosomal contacts at BMI and SCZ risk sequences [24, 28]. These analyses resulted in additional evidence for cell-specific regulation for disease-associated variants (Additional file 2: Table S9). These include, for example, BMI and SCZ risk SNPs in *PRKD1*, encoding a protein kinase important for dopaminergic neuron oxidative stress-survival [76], or dopa-decarboxylase *DDC*, also known as aromatic L-amino acid decarboxylase (*AADC*) essential for dopamine and serotonin synthesis [77] which via intra-chromosomal conformations is connected to intra-genic sequences of the actin nucleator *COBL* essential for neurite induction and branching [78] (Additional file 3: Figure S11). Because each of the two computational approaches to assess chromosomal contacts (HICCUPS and locus-specific binomial statistics) provided evidence for an interaction between SCZ and BMI risk sequences in the MDN 3DG, we decided to pursue additional, cell type-specific, and unbiased (genome-scale) approaches to gain deeper understanding of the role of the spatial genome in governing the convergence of SCZ and BMI genomic risk architectures.

### Spatial genome modeling reveals Euclidean hot spots of risk-associated chromatin domains enriched for dopaminergic signaling, lipid metabolism, and reward pathways

Next, we reconstructed the three-dimensional spatial genome with the MDN-specific ^*Tn5*^Hi-C dataset, to visualize the nuclear topography and Hi-C interaction frequencies of SCZ and BMI risk loci within their respective chromatin domains. Using chrom3D-based Monte Carlo simulations, we computed domain-domain interactions from the ^*Tn5*^Hi-C contact matrix, taking into account spatial constraints from intra- and inter-chromosomal interaction scores and polymer physics [55, 79]. Chrom3D domains were called in Arrowhead at 50 kb resolution, resulting in *N* = 3066 chromatin bead domains called for the diploid genome (of the MDN, averaging 1.3 Mb in length (Fig. 2a, c, and Additional file 3: Figure S2).

We reasoned that MDN chromatin domains harboring risk sequences both for schizophrenia and BMI could serve as useful “anchors” to map chromosomal interactions across the genetic risk architectures of both these conditions. We counted 100 domains in the diploid MDN genome sharing GWAS risk sequences both from a SNP-based BMI GWAS summary table [48] and from a linkage disequilibrium/risk locus-defined schizophrenia GWAS summary table [56]. The schizophrenia risk loci average in length at 256 kb± 749 (Additional file 2: Table S4; Additional file 3: Figure S3). Altogether, 43% or 60/139 schizophrenia risk loci and the 31% or 90/289 BMI SNPs are located within the “shared” 100 domains of our chrom3D-computed MDN spatial genome, harboring 585 intra- and 198 *trans*-chromosomal contacts interconnecting BMI SNPs with SCZ risk loci (Fig. 2d). These effects were highly specific for risk domains shared between BMI and SCZ, because both these two conditions, separately, ranked top in shared domain enrichment as assessed by permutational analysis on Nurr1^+^/NeuN^+^ MDN domains, conducted with 28 different GWAS datasets representing different medical and psychiatric traits (Additional file 3: Figure S4).

Interestingly, random shuffling using the 3066 MDN domains as background results in a significantly higher number of shared risk MDN domains as compared to the 100 that were observed for this cell type (Additional file 3: Figure S3). Next, we calculated Euclidean pairwise distances among the 100 chromatin domains (also called “beads” in the chrom3D toolkit [55]) (Fig. 2c). There were up to 11 clusters, each comprised of multiple domains that (1) harbor both BMI and SCZ GWAS risk sequence and (2) are confined in close proximity within the 3D space of the nucleus. We refer to these clusters as Euclidean “hot spots” (referred to as “EH” hereafter), with each EH called at *p* < 3.96 × 10^−6^ to 0.01 compared to random shuffling of the 100 shared domains (see Additional file 3: Figure S2) in the MDN spatial genome, each comprised of a specific set of multiple domains/beads tight together, with the smallest and largest cluster comprised of 3 and 13 beads, respectively (Fig. 2d). Note that 23/100 domains participated in connecting six EHs (labeled A–D in Fig. 2d heatmap), while 77/100 shared domains were confined to a single EH. We note that while the majority of clusters are defined by diploid contributions, a subset thereof including EHs no. 1, no. 5, and no. 10 show allelic imbalance (Additional file 2: Table S4). Future work will be required in order to understand the allelic bias in chrom3D-based spatial genome modeling. Strikingly, however, the 11 EHs, comprised of chromatin domains shared by BMI and schizophrenia GWAS, include 339 risk genes with functional enrichment for lipid regulation, axon guidance and dopaminergic signaling, reward and addiction pathways, starvation response, and signaling cascades linked to BDNF, a neurotrophic factor representing a key molecule for synaptic plasticity and regulation of food intake and body weight by modulating MDN activity including their (dopaminergic) fiber projections into the forebrain [80] (Fig. 2e, f, Additional file 2: Table S7).

In order to better understand the regulatory elements orchestrating these EH-associated gene groups highly relevant for MDN functions, we calculated, within each of the 11 EHs, the number of chromosomal conformations interconnecting BMI and SCZ risk sequences, as indicated in Fig. 2d, and their intersection with a database on transcribed regulatory non-coding sequence generated from MDN somata that had been laser-dissected from adult human midbrain [8]. We counted, at 40 kb resolution, a total of 333 actively transcribed non-coding elements, using a list of 70,996 MDN transcribed sequences [8] as input. These 333 sequences included altogether 251 regulatory motifs (see the “Methods” section). In remarkable agreement with the aforementioned gene-based GO analyses, these 225 regulatory motifs were enriched for white fat cell differentiation and lipid regulation, dopaminergic neurogenesis and neural fate commitment, and SMAD transcription factors implicated in cholesterol metabolism, reward and addiction [81, 82], and dopaminergic neuron health and survival [83] (Fig. 2g, h, Additional file 2: Table S6). Remarkably, the EH-associated genes and motifs showed significant protein-protein interaction network effects, including an extended transcription factor network interconnected to disease-relevant ion channels and receptors (incl. CACNA1D/I and DRD2 dopamine receptor), to NCAM1 and other cell adhesion molecules and to key orchestrators of cell metabolism and body weight, including RPTOR, MTOR, and PPARG (Fig. 2g, h).

**Fig. 3:**
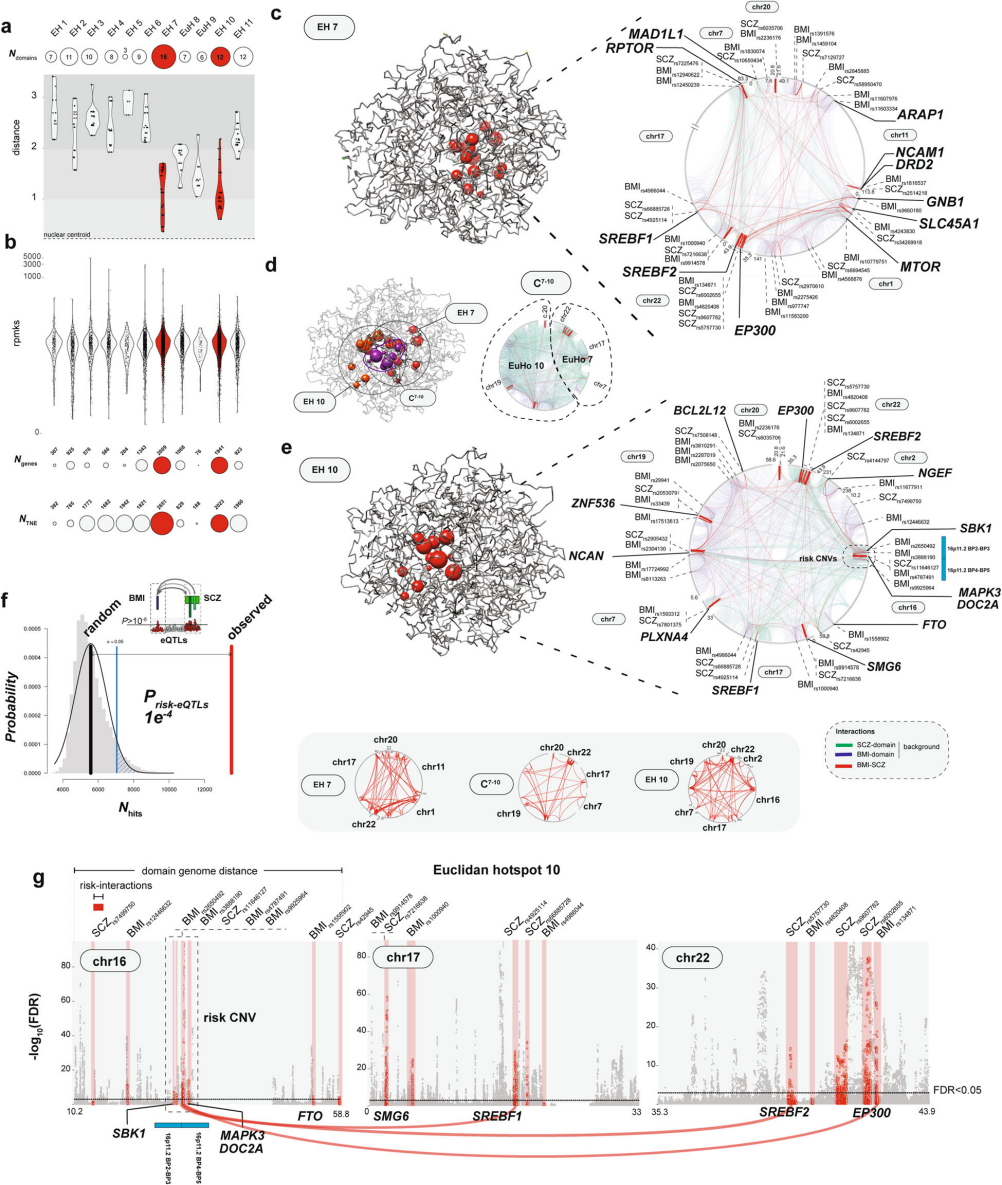
Euclidean hot spot analysis. **a** Violin plot representing each of the 11 EH, showing distance of domain-to-nuclear centroid. Scaled circles represent the number of domains found in each EH. Red color represents EHs no.7 and no.10 harboring the largest number of domains, as indicated. **b** Violin plot representing rpmks gene expression levels per EH, circle plots showing number of genes (*N*_genes_) and number of transcribed non-coding elements (*N*_TNE_) per EH, as indicated. Note that EH no. 7 and no. 10 (red) represent transcriptionally active domain clusters. **c–e** In silico chrom3D models of MDN spatial genome, red beads = EH-specific domains, **c** EH no. 7, **d** pink beads = cluster C (from Fig. 2e) domains shared among EH no. 7 and no. 10, and **e** EH no. 10. Circos plot interactomes for (**c**, right panel) EH no.7 and (**d**, right panel) cluster C^(7,10)^ and (**e**, right panel) EH no. 10, showing for each participating chromosome the location of (red tick marks) risk SNPs and (red lines) Hi-C Pro called chromosomal contacts reciprocally interconnecting BMI-to-SCZ risk variants at 40 kb resolution, including position of selected target genes. “Background chromosomal contacts” (blue) mark “BMI risk variant-to-rest of EH” contacts and (green) “SCZ risk variant-to-rest of EH” contacts, using BMI and SCZ index SNPs (Additional file 2: Table S4). **f** Permutation analysis probability density plot. The likelihood of cross disorder BMI-to-SCZ reciprocal interactions associated to significant brain *cis*-eQTLs (called at FDR < 10^−8^) was performed by comparing the association of randomized cross-disorder interactions (10,000 permutations) over the observed overlap. **g** Representative brain *cis*-eQTLs Manhattan plots shown for three domains from EH no. 10. Red shaded fields mark sequences fulfilling each of the following three conditions: (i) harboring both SCZ and BMI risk polymorphisms, (ii) anchored in cross-disorder chromosomal contact within the EH, and (iii) harboring significant brain *cis*-eQTLs. As an example, highlighted by red connector lines scaled to the ICED interaction frequency, interactions anchored in chr1611p2B2-B3/B4-B5 locus implicated in weight regulation and neurodevelopment [87] to disease-relevant associated genes *SREBF1*, *SREBF2*, and *EP300*

Consistent with the general notion that gene expression activity within specific chromosomal loci is much lower towards the nuclear periphery as compared to more central positioning inside the nucleus [79, 84–86], expression of genes and of non-coding regulatory elements in our risk-associated chromatin domains show moderate anti-correlation with domain-to-centroid distance (*R*~ − 0.30) (Additional file 3: Figure S12). Furthermore, chrom3D modeling using Hi-C datasets from nine different cell types, including Ngn2-differentiated glutamatergic neurons, and the fetal cortical plate which is overwhelmingly comprised of neurons, showed that the most centrally located Euclidean hot spots in the dopaminergic neurons, including E^H7^ and E^H10^, showed strong cell type-specific regulation with dopamine neurons showing for these hot spots the shortest distances to the centroid and between the individual domains (Additional file 3: Figure S13). Importantly, distances to centroid, which in the chrom3D model for E^H7^ and E^H10^ was resolved differentially for an “A” and a “B” haplotype, were indistinguishable for maps generated for Nurr1^+^/NeuN^+^ MDN using our transposase-based ^Tn5^Hi-C protocol and chrom3D maps that we generated for Nurr1^+^/NeuN^+^ MDN using the commercially available ^Arima^Hi-C kit. These findings, taken together, suggest that each of the 11 EHs could serve as locus-specific “connectivity hub” linking disease-relevant genes to key molecules associated with dopaminergic signaling. As an example, Fig. 3a, b shows EHs no. 7 and 10 which both extend deep into the nuclear interior harboring the highest number of expressed genes and non-coding regulatory elements. In addition, several domains link these EHs close to the nuclear centroid, forming the C^(7,10)^ interconnection (Fig. 3c–e). Within EH no. 7, ^*Tn5*^Hi-C interacting domains from chrs. 1, 7, 11, 20, and 22 interconnect to one of the top-scoring schizophrenia risk loci, *MAD1L1* [56], which in context of reward-associated paradigms is associated with significant functional hypoactivation of the ventral midbrain and its prefrontal targets [88], to multiple genes each located within 40 kb from both BMI and SCZ risk sequences, such as, (i) chr. 11 *DRD2* dopamine receptor, a critical antipsychotic drug target [89], (ii) chr. 1 *GBN1* neurodevelopmental risk gene encoding a guanine-nucleotide binding protein coupled to dopamine receptor systems [90], (iii) chrs. 17 and 22 *SREBF1* and *SREBF2* transcription factors highly important for cholesterol and fatty acid biosynthesis including antipsychotic drug-induced metabolic side effects [91, 92], and (iv) chrs. 1 and 17 *RPTOR* and *MTOR* genes, two key members in a nutrient-sensitive pathway controlling cell growth [93] (Fig. 3c). Likewise, in EH no. 10, SCZ and BMI risk sequences from domains in chrs. 2, 7, 16, 17, 19, 20, and 22 are interconnected with 16p11.2 neurodevelopmental risk sequences often affected by micro-deletions and -duplications associated with obesity or underweight phenotypes, micro- and macrocephaly in conjunction with symptoms on the autism and psychosis spectrum [87, 94] (Fig. 3e).

Next, we mapped the distribution of expression quantitative trait loci (eQTLs), using the collection of 643,032 *cis*-eQTL (FDR corrected *p* < 10e^−8^) calculated from SNP–gene pairs within 1 Mb of a gene, generated from *N* = 467 brain-specific RNA-seq datasets [62]. We counted within the 11 EHs 13,575 *cis*-eQTLs associated with chromosomal contacts interconnecting BMI and SCZ risk sequences, representing a significant enrichment when compared against the background of all EH-specific contacts (*p* = 10e^−4^ with 10,000 permutations) (Fig. 3f). Because many eQTL association results may not be independent but instead result from high linkage disequilibrium between eQTL SNPs, we performed clumping for each gene using the PLINK 1.90 software [95] at a clump distance of 250 kb and an *r*^2^ of 0.2 and 0.6. The clumped summary statistics confirmed significant eQTL enrichment within the 11 Euclidean hot spots (Additional file 3: Figure S14). As a representative example, Fig. 3g shows a subset of SCZ and BMI risk eQTL-bound chromosomal contacts, interconnecting the aforementioned 16p11.2 neurodevelopmental/obesity copy number variant locus with numerous metabolic regulators positioned in domains of chrs. 17 and 22. In addition, we screened our collection of risk-associated chromosomal contacts within the 11 EHs (Fig. 2f) against the Genotype-Tissue Expression Project (GTEx) eQTL resource and observed, against the genome-wide background of the entire collection of tissue-specific eQTLs, significant enrichments (*p* < 0.05) for the brain and for adipocyte-dominated tissues (Additional file 3: Figure S14).

## Discussion

Here, we map chromosomal conformations and model their three-dimensional intra-nuclear positioning in adult midbrain dopaminergic neurons (MDN). This cell type is critically involved both in ventral forebrain circuitries regulating eating behavior and metabolism [5, 6] and dorsal forebrain circuitries sub-serving cognition and complex behaviors [96]. Therefore, elucidating the genomic and epigenomic profiles of this group of neurons is extremely important for a deeper understanding of the pathophysiology of schizophrenia and its co-occurring comorbidities including metabolic disorders which is estimated to affect one of three patients [11]. The underlying causes resulting in such high rates of metabolic disease in subjects with schizophrenia remain incompletely understood and are likely to include both medication-specific [97] and medication-independent factors [98]. Unfortunately, while there is circumstantial evidence for a role of immune and endocrine regulators operating in the context of atypical antipsychotic medication and other well established risk factors for metabolic syndrome [13], extremely little is known about the role of the MDN. Obesity status is associated with neurochemical alterations in human MDN, affecting expression of dopamine receptors and transporters [99]. As described above, based on our chromosomal conformation mappings, there are at least 11 “Euclidean hot spots” of clustered chromatin domains with increased interaction frequencies of risk sequences for SCZ and BMI. Within these 11 “EHs,” inter- and intra-chromosomal contacts interconnecting SCZ and BMI risk sequences showed significant enrichment for brain-specific expression quantitative trait loci (eQTL), with gene ontologies and regulatory motifs related to adipogenesis, dopaminergic neurogenesis and signaling, and nicotine and reward/addiction-related pathways. These include, among others, established “triple” regulators governing feeding behavior and social cognition and antipsychotic response profiles. These include, to mention just three examples, the *DRD2* [100] dopamine receptor, the *SREBF* (*1*/*2*) transcription factor family encoding sterol regulatory element-binding proteins (SREBPs) serving as key control points for lipid metabolism [101], and cell-autonomous fatty acid synthesis essential for proper dendritic arborization in central neurons [102], and sequences in the 16p11.2 neurodevelopmental risk locus [87, 94]. Therefore, it is extremely interesting that genetic polymorphism in the *SRBF* genes are associated with genetic risk both for BMI and SCZ and via EH *trans*-chromosomal contact in physical interactions with other key loci including *DRD2* (Fig. 3c–e). Interestingly, *SRBF* risk allele carriers are affected by metabolic syndrome in combination with impaired cognitive processing, as compared to subjects with schizophrenia not carrying the risk allele [103]. Future studies, using the resource generated here, including the list of 783 chromosomal contacts interconnecting BMI risk SNPs with SCZ risk loci in MDN, often with multiple genes involved (Additional file 2: Table S5), should examine in the translational model whether the genomic or epigenomic editing of cross-disorder gene targets, including parallel mobilization of multiple chromosomal loci to specific nuclear subcompartments such as lamina-associated heterochromatin or transcriptional hubs including Cajal bodies [104], will affect cognition and metabolism in the animal.

Additional use of our resource may arise in the context of “personalized medicine” and targeted refinement of the population, including polygenic risk scores (PRS) subsetting and predicting disease liability and treatment response based on individual genotype. This is especially important given that a subset of antipsychotic drugs could act as major driver in the incidence of metabolic syndrome in psychiatric populations [13]. However, given the very limited overlap between SCZ and BMI genetic risk architectures, at least when common variant GWAS loci [18, 19] are compared, epigenomic approaches may surmount this constraint, by considering the physical interactions of risk alleles in a cell type of interest, as an alternative to the PRS construct, modeled as a quantitative composite of weighted risk alleles on the linear genome scale. Thus, our spatial genome and transcriptome resource derived from adult dopaminergic midbrain neurons, modeling the interaction of distant genomic regions, may be particularly useful for the study of co-morbid conditions involving psychosis and metabolic syndrome and obesity.

Our studies provide proof of concept that genome-scale chromosome conformation mapping, at least on the scale of chromatin domains in the kilo- to megabase range, is feasible even in rare cell populations extracted from the human postmortem brain. However, while a systematic comparison of the various Hi-C protocols would go beyond the scope of the present study, it is important to discuss the advantages and limitations of Hi-C protocols applicable to limited amounts of input material, such as 5–10 × 10^3^ nuclei that had undergone immunotagging and FACS sorting and separation prior to the Hi-C procedure. Our ^*Tn5*^Hi-C protocol for example involves fewer steps when compared to conventional Hi-C procedures and some of its recently introduced derivatives such as DNase Hi-C [105], owing to transposase 5 (Tn5)-based tagmentation. However, the trade-off of Hi-C libraries produced from such small numbers of postmortem brain nuclei, as compared to conventional Hi-C libraries prepared from two to three orders of magnitude larger numbers of brain nuclei, is reflected by poorer quality indices such as the cis/trans (c/t) contact ratio (N libraries, mean ± S.D.: conventional Hi-C *N* = 8, c/t 3.41 ± 0.82; ^*Tn5*^Hi-C *N* = 12, c/t 1.10 ± 0.67; ^Arima^HiC *N* = 2 c/t 4.66 and 1.11; Additional file 2: Table S1) or the proportion of valid interaction pairs (N libraries, mean ± S.D.: conventional Hi-C N = 8, val. 0.80 ± 0.08; ^*Tn5*^Hi-C N = 12, val. 0.18 ± 0.12; ^Arima^HiC N = 2 val. 0.32 and 0.39; Additional file 2: Table S1). As a result, deeper sequencing may be required to compensate at least partially for an overall decrease in yield. Of note, in freshly harvested cells, Tn5 tagmentation has been used recently in scaled-up single-cell Hi-C protocols designed to process thousands of individual nuclei in parallel [106]. However, it remains to be tested in postmortem tissue, including single-MDN nuclei, whether the contact map constructed from pooled single-cell Hi-C datasets offers advantages over the ensemble-based Hi-C dataset generated here. Finally, it is important to note that our ^*Tn5*^Hi-C (just like any other Hi-C) contact map, including the Euclidean hot spots analyzed here, ultimately represent contact frequencies, not actual spatial proximities. However, it has been suggested that Hi-C and chromosome conformation capture, on mega-domain scales ranging from ~ 300 kb to 10 Mb, largely is correlated with spatial distances as determined by DNA FISH [107], but this remains to be examined for the hot spots and clustered domains discussed here. However, dynamic modeling of interphase chromosome organization, including the “loop extrusion model” affecting formation and spatial proximity of TADs and larger chromatin domains, has shown that inter-domain functional (CF) and structural (spatial proximity) measurements are distinct, with limited potential for cross-validation [108].

Finally, our study resolves the apparent paradox that transcriptome mappings in conjunction with stratified LD score regression have assigned to brain high enrichment scores both for BMI and psychiatric disorders including SCZ [109], yet there is very limited “cross-disorder” overlap or proximity of the disease-relevant sequence variants and polymorphisms [18, 19]. Based on the extensive web of cross-disorder chromosomal contacts shown here for the MDN as key neuronal population regulating cognition and metabolism, we predict that spatial genome mappings in specific cell populations directly extracted from human brain tissue will provide novel and unprecedented insights into the genomic architecture of medical and psychiatric comorbidities of tremendous public health importance. These co-morbidities include psychosis and metabolic syndrome, narcotics addiction and pain, chronic alcoholism and cognitive decline, among many others.

## Conclusions

The present study demonstrates, with two independent experimental protocols, that Hi-C spatial genome mapping is feasible from a limited number of FACS-sorted nuclei from postmortem brain tissue. This will allow for cell type-specific 3D genome mapping from some of the brain’s rare cell populations such as the monoaminergic cell groups residing in the basal forebrain and in the mid- and hindbrain. We generated Hi-C maps from ensembles of ventral midbrain Nurr1^+^/NeuN^+^ dopaminergic neuron nuclei and discovered that some of the chromosomal conformations harboring common variants associated with risk for schizophrenia are, in non-random manner, co-localized with chromosomal domains harboring risk variants associated with excess body mass. These genomic interactions included at least 11 “Euclidean hot spots” with inter- and intra-chromosomal contacts interconnecting SCZ and BMI risk sequences significantly enriched for brain-specific expression quantitative trait loci (eQTL), with gene ontologies and regulatory motifs related to adipogenesis, dopaminergic neurogenesis and signaling, and nicotine and reward/addiction-related pathways. More broadly, the 3D genome-based concepts presented here are of interest for other medical co-morbidities for which the respective genetic risk architectures show only very limited to no cross-disorder overlap.

## Supplementary information

**Additional file 1.** Supplemental Methods.

**Additional file 2.** Supplementary Tables.

**Additional file 3.** Supplementary Figures.

**Additional file 4.** Datafile (txt), QTL analysis 1.

**Additional file 5.** Datafile (txt), QTL analysis 2.

## Data Availability

Datasets generated during the course of this project are available in the following Synapse repositories: syn20833047 (midbrain RNA-seq and HiC datasets) [110] and syn20545534 and https://genome.ucsc.edu/s/sespeso/EspesoGil_Halene2019 [111]. The following datasets, from previous publications, were downloaded for analyses: syn12979101 (https://www.synapse.org/#!Synapse:syn12979101, registration required) [112], GSE63525 (https://www.ncbi.nlm.nih.gov/geo/query/acc.cgi?acc=GSE63525 [113], GSE63525 (https://www.ncbi.nlm.nih.gov/geo/query/acc.cgi?acc=GSE63525), and phs001190.v1.p1 (https://www.ncbi.nlm.nih.gov/projects/gap/cgi-bin/study.cgi?study_id=phs001190.v1.p1) [114].

## References

[1] Hegarty SV, Sullivan AM, O'Keeffe GW (2013). Midbrain dopaminergic neurons: a review of the molecular circuitry that regulates their development. Dev Biol.

[2] German DC, Schlusselberg DS, Woodward DJ (1983). Three-dimensional computer reconstruction of midbrain dopaminergic neuronal populations: from mouse to man. J Neural Transm.

[3] Pakkenberg B, Moller A, Gundersen HJ, Mouritzen Dam A, Pakkenberg H (1991). The absolute number of nerve cells in substantia nigra in normal subjects and in patients with Parkinson’s disease estimated with an unbiased stereological method. J Neurol Neurosurg Psychiatry.

[4] Grace AA (2016). Dysregulation of the dopamine system in the pathophysiology of schizophrenia and depression. Nat Rev Neurosci.

[5] Lindgren E, Gray K, Miller G, Tyler R, Wiers CE, Volkow ND, Wang GJ (2018). Food addiction: a common neurobiological mechanism with drug abuse. Front Biosci.

[6] Rui L (2013). Brain regulation of energy balance and body weight. Rev Endocr Metab Disord.

[7] Palmiter RD (2007). Is dopamine a physiologically relevant mediator of feeding behavior?. Trends Neurosci.

[8] Dong X, Liao Z, Gritsch D, Hadzhiev Y, Bai Y, Locascio JJ, Guennewig B, Liu G, Blauwendraat C, Wang T (2018). Enhancers active in dopamine neurons are a primary link between genetic variation and neuropsychiatric disease. Nat Neurosci.

[9] Subramaniam M, Lam M, Guo ME, He VY, Lee J, Verma S, Chong SA (2014). Body mass index, obesity, and psychopathology in patients with schizophrenia. J Clin Psychopharmacol.

[10] Pillinger T, Beck K, Gobjila C, Donocik JG, Jauhar S, Howes OD (2017). Impaired glucose homeostasis in first-episode schizophrenia: a systematic review and meta-analysis. JAMA Psychiatry.

[11] Mitchell AJ, Vancampfort D, Sweers K, van Winkel R, Yu W, De Hert M (2013). Prevalence of metabolic syndrome and metabolic abnormalities in schizophrenia and related disorders--a systematic review and meta-analysis. Schizophr Bull.

[12] Monteleone P, Martiadis V, Maj M (2009). Management of schizophrenia with obesity, metabolic, and endocrinological disorders. Psychiatr Clin North Am.

[13] Penninx B, Lange SMM (2018). Metabolic syndrome in psychiatric patients: overview, mechanisms, and implications. Dialogues Clin Neurosci.

[14] Lee EE, Liu J, Tu X, Palmer BW, Eyler LT, Jeste DV (2018). A widening longevity gap between people with schizophrenia and general population: a literature review and call for action. Schizophr Res.

[15] Laursen TM, Nordentoft M, Mortensen PB (2014). Excess early mortality in schizophrenia. Annu Rev Clin Psychol.

[16] Li KJ, Greenstein AP, Delisi LE (2018). Sudden death in schizophrenia. Curr Opin Psychiatry.

[17] Postolache TT, Del Bosque-Plata L, Jabbour S, Vergare M, Wu R, Gragnoli C (2019). Co-shared genetics and possible risk gene pathway partially explain the comorbidity of schizophrenia, major depressive disorder, type 2 diabetes, and metabolic syndrome. Am J Med Genet B Neuropsychiatr Genet.

[18] Bulik-Sullivan BK, Loh PR, Finucane HK, Ripke S, Yang J, Patterson N, Daly MJ, Price AL, Neale BM, Schizophrenia Working Group of the Psychiatric Genomics C (2015). LD score regression distinguishes confounding from polygenicity in genome-wide association studies. Nat Genet.

[19] Zheutlin AB, Dennis J, Linnér RK, Moscati A, Restrepo N, Straub P, Ruderfer D, Castro VM, Chen C-Y, Ge T, et al. Penetrance and pleiotropy of polygenic risk scores for schizophrenia in 106,160 patients across four healthcare systems. Am J Psychiatry. 2019;176:846–55. 10.1176/appi.ajp.2019.18091085 PMC6961974 31416338

[20] Bahrami S, Steen NE, Shadrin A, O’Connell K, Frei O, Bettella F, Wirgenes KV, Krull F, Fan CC, Dale AM, et al. Shared genetic loci between body mass index and major psychiatric disorders: a genome-wide association study. JAMA Psychiatry. 2020. 10.1001/jamapsychiatry.2019.4188 PMC6990967 31913414

[21] Dekker J, Marti-Renom MA, Mirny LA (2013). Exploring the three-dimensional organization of genomes: interpreting chromatin interaction data. Nat Rev Genet.

[22] Babaei S, Mahfouz A, Hulsman M, Lelieveldt BP, de Ridder J, Reinders M (2015). Hi-C chromatin interaction networks predict co-expression in the mouse cortex. PLoS Comput Biol.

[23] Jiang Y, Loh YE, Rajarajan P, Hirayama T, Liao W, Kassim BS, Javidfar B, Hartley BJ, Kleofas L, Park RB (2017). The methyltransferase SETDB1 regulates a large neuron-specific topological chromatin domain. Nat Genet.

[24] Won H, de la Torre-Ubieta L, Stein JL, Parikshak NN, Huang J, Opland CK, Gandal MJ, Sutton GJ, Hormozdiari F, Lu D (2016). Chromosome conformation elucidates regulatory relationships in developing human brain. Nature.

[25] Giusti-Rodriguez PM, Sullivan PF. Schizophrenia and a high-resolution map of the three-dimensional chromatin interactome of adult and fetal cortex. bioRxiv. 2018:406330.

[26] Wang Daifeng, Liu Shuang, Warrell Jonathan, Won Hyejung, Shi Xu, Navarro Fabio C. P., Clarke Declan, Gu Mengting, Emani Prashant, Yang Yucheng T., Xu Min, Gandal Michael J., Lou Shaoke, Zhang Jing, Park Jonathan J., Yan Chengfei, Rhie Suhn Kyong, Manakongtreecheep Kasidet, Zhou Holly, Nathan Aparna, Peters Mette, Mattei Eugenio, Fitzgerald Dominic, Brunetti Tonya, Moore Jill, Jiang Yan, Girdhar Kiran, Hoffman Gabriel E., Kalayci Selim, Gümüş Zeynep H., Crawford Gregory E., Roussos Panos, Akbarian Schahram, Jaffe Andrew E., White Kevin P., Weng Zhiping, Sestan Nenad, Geschwind Daniel H., Knowles James A., Gerstein Mark B. (2018). Comprehensive functional genomic resource and integrative model for the human brain. Science.

[27] Rhie SK, Schreiner S, Witt H, Armoskus C, Lay FD, Camarena A, Spitsyna VN, Guo Y, Berman BP, Evgrafov OV (2018). Using 3D epigenomic maps of primary olfactory neuronal cells from living individuals to understand gene regulation. Sci Adv.

[28] Rajarajan Prashanth, Borrman Tyler, Liao Will, Schrode Nadine, Flaherty Erin, Casiño Charlize, Powell Samuel, Yashaswini Chittampalli, LaMarca Elizabeth A., Kassim Bibi, Javidfar Behnam, Espeso-Gil Sergio, Li Aiqun, Won Hyejung, Geschwind Daniel H., Ho Seok-Man, MacDonald Matthew, Hoffman Gabriel E., Roussos Panos, Zhang Bin, Hahn Chang-Gyu, Weng Zhiping, Brennand Kristen J., Akbarian Schahram (2018). Neuron-specific signatures in the chromosomal connectome associated with schizophrenia risk. Science.

[29] Mah W, Won H. The three-dimensional landscape of the genome in human brain tissue unveils regulatory mechanisms leading to schizophrenia risk. Schizophr Res. 2019. 10.1016/j.schres.2019.03.007 PMC6748876 30894290

[30] Rao SS, Huntley MH, Durand NC, Stamenova EK, Bochkov ID, Robinson JT, Sanborn AL, Machol I, Omer AD, Lander ES, Aiden EL (2014). A 3D map of the human genome at kilobase resolution reveals principles of chromatin looping. Cell.

[31] van Berkum NL, Lieberman-Aiden E, Williams L, Imakaev M, Gnirke A, Mirny LA, Dekker J, Lander ES. Hi-C: a method to study the three-dimensional architecture of genomes. J Vis Exp. 2010. 10.3791/1869 PMC3149993 20461051

[32] Giusti-Rodríguez P, Lu L, Yang Y, Crowley CA, Liu X, Juric I, Martin JS, Abnousi A, Allred SC, Ancalade N, et al: Using three-dimensional regulatory chromatin interactions from adult and fetal cortex to interpret genetic results for psychiatric disorders and cognitive traits. bioRxiv. 2019:406330.

[33] Di Lorenzo Alho AT, Suemoto CK, Polichiso L, Tampellini E, de Oliveira KC, Molina M, Santos GA, Nascimento C, Leite RE, de Lucena Ferreti-Rebustini RE (2016). Three-dimensional and stereological characterization of the human substantia nigra during aging. Brain Struct Funct.

[34] Bjorklund A, Dunnett SB (2007). Dopamine neuron systems in the brain: an update. Trends Neurosci.

[35] Azevedo FA, Carvalho LR, Grinberg LT, Farfel JM, Ferretti RE, Leite RE, Jacob Filho W, Lent R, Herculano-Houzel S (2009). Equal numbers of neuronal and nonneuronal cells make the human brain an isometrically scaled-up primate brain. J Comp Neurol.

[36] von Bartheld CS, Bahney J, Herculano-Houzel S (2016). The search for true numbers of neurons and glial cells in the human brain: a review of 150 years of cell counting. J Comp Neurol.

[37] Rice PA, Baker TA (2001). Comparative architecture of transposase and integrase complexes. Nat Struct Biol.

[38] Dobin A, Davis CA, Schlesinger F, Drenkow J, Zaleski C, Jha S, Batut P, Chaisson M, Gingeras TR (2013). STAR: ultrafast universal RNA-seq aligner. Bioinformatics.

[39] DeLuca DS, Levin JZ, Sivachenko A, Fennell T, Nazaire MD, Williams C, Reich M, Winckler W, Getz G (2012). RNA-SeQC: RNA-seq metrics for quality control and process optimization. Bioinformatics.

[40] Risso D, Ngai J, Speed TP, Dudoit S (2014). Normalization of RNA-seq data using factor analysis of control genes or samples. Nat Biotechnol.

[41] Poulin JF, Zou J, Drouin-Ouellet J, Kim KY, Cicchetti F, Awatramani RB (2014). Defining midbrain dopaminergic neuron diversity by single-cell gene expression profiling. Cell Rep.

[42] Mancarci B. Ogan, Toker Lilah, Tripathy Shreejoy J., Li Brenna, Rocco Brad, Sibille Etienne, Pavlidis Paul (2017). Cross-Laboratory Analysis of Brain Cell Type Transcriptomes with Applications to Interpretation of Bulk Tissue Data. eneuro.

[43] de Leeuw CA, Mooij JM, Heskes T, Posthuma D (2015). MAGMA: generalized gene-set analysis of GWAS data. PLoS Comput Biol.

[44] Schizophrenia Working Group of the Psychiatric Genomics C (2014). Biological insights from 108 schizophrenia-associated genetic loci. Nature.

[45] Lambert JC, Ibrahim-Verbaas CA, Harold D, Naj AC, Sims R, Bellenguez C, DeStafano AL, Bis JC, Beecham GW, Grenier-Boley B (2013). Meta-analysis of 74,046 individuals identifies 11 new susceptibility loci for Alzheimer’s disease. Nat Genet.

[46] Demontis D, Walters RK, Martin J, Mattheisen M, Als TD, Agerbo E, Baldursson G, Belliveau R, Bybjerg-Grauholm J, Baekvad-Hansen M (2019). Discovery of the first genome-wide significant risk loci for attention deficit/hyperactivity disorder. Nat Genet.

[47] Okbay A, Baselmans BM, De Neve JE, Turley P, Nivard MG, Fontana MA, Meddens SF, Linner RK, Rietveld CA, Derringer J (2016). Genetic variants associated with subjective well-being, depressive symptoms, and neuroticism identified through genome-wide analyses. Nat Genet.

[48] Locke AE, Kahali B, Berndt SI, Justice AE, Pers TH, Day FR, Powell C, Vedantam S, Buchkovich ML, Yang J (2015). Genetic studies of body mass index yield new insights for obesity biology. Nature.

[49] Liu JZ, van Sommeren S, Huang H, Ng SC, Alberts R, Takahashi A, Ripke S, Lee JC, Jostins L, Shah T (2015). Association analyses identify 38 susceptibility loci for inflammatory bowel disease and highlight shared genetic risk across populations. Nat Genet.

[50] Nikpay M, Goel A, Won HH, Hall LM, Willenborg C, Kanoni S, Saleheen D, Kyriakou T, Nelson CP, Hopewell JC (2015). A comprehensive 1,000 genomes-based genome-wide association meta-analysis of coronary artery disease. Nat Genet.

[51] Wood AR, Esko T, Yang J, Vedantam S, Pers TH, Gustafsson S, Chu AY, Estrada K, Luan J, Kutalik Z (2014). Defining the role of common variation in the genomic and biological architecture of adult human height. Nat Genet.

[52] Auton A, Brooks LD, Durbin RM, Garrison EP, Kang HM, Korbel JO, Marchini JL, McCarthy S, McVean GA, Abecasis GR, Genomes Project C (2015). A global reference for human genetic variation. Nature.

[53] Servant N, Varoquaux N, Lajoie BR, Viara E, Chen CJ, Vert JP, Heard E, Dekker J, Barillot E (2015). HiC-Pro: an optimized and flexible pipeline for Hi-C data processing. Genome Biol.

[54] Durand NC, Shamim MS, Machol I, Rao SS, Huntley MH, Lander ES, Aiden EL (2016). Juicer provides a one-click system for analyzing loop-resolution Hi-C experiments. Cell Syst.

[55] Paulsen J, Liyakat Ali TM, Collas P (2018). Computational 3D genome modeling using Chrom3D. Nat Protoc.

[56] Pardinas AF, Holmans P, Pocklington AJ, Escott-Price V, Ripke S, Carrera N, Legge SE, Bishop S, Cameron D, Hamshere ML (2018). Common schizophrenia alleles are enriched in mutation-intolerant genes and in regions under strong background selection. Nat Genet.

[57] Corradin O, Cohen AJ, Luppino JM, Bayles IM, Schumacher FR, Scacheri PC (2016). Modeling disease risk through analysis of physical interactions between genetic variants within chromatin regulatory circuitry. Nat Genet.

[58] Whalen S, Pollard KS (2019). Most chromatin interactions are not in linkage disequilibrium. Genome Res.

[59] Delaneau O., Zazhytska M., Borel C., Giannuzzi G., Rey G., Howald C., Kumar S., Ongen H., Popadin K., Marbach D., Ambrosini G., Bielser D., Hacker D., Romano L., Ribaux P., Wiederkehr M., Falconnet E., Bucher P., Bergmann S., Antonarakis S. E., Reymond A., Dermitzakis E. T. (2019). Chromatin three-dimensional interactions mediate genetic effects on gene expression. Science.

[60] Servant N, Lajoie BR, Nora EP, Giorgetti L, Chen CJ, Heard E, Dekker J, Barillot E (2012). HiTC: exploration of high-throughput ‘C’ experiments. Bioinformatics.

[61] Finucane HK, Bulik-Sullivan B, Gusev A, Trynka G, Reshef Y, Loh PR, Anttila V, Xu H, Zang C, Farh K (2015). Partitioning heritability by functional annotation using genome-wide association summary statistics. Nat Genet.

[62] Fromer M, Roussos P, Sieberts SK, Johnson JS, Kavanagh DH, Perumal TM, Ruderfer DM, Oh EC, Topol A, Shah HR (2016). Gene expression elucidates functional impact of polygenic risk for schizophrenia. Nat Neurosci.

[63] Bindea G, Mlecnik B, Hackl H, Charoentong P, Tosolini M, Kirilovsky A, Fridman WH, Pages F, Trajanoski Z, Galon J (2009). ClueGO: a Cytoscape plug-in to decipher functionally grouped gene ontology and pathway annotation networks. Bioinformatics.

[64] Jiang Y, Matevossian A, Huang HS, Straubhaar J, Akbarian S (2008). Isolation of neuronal chromatin from brain tissue. BMC Neurosci.

[65] Matevossian A, Akbarian S. Neuronal nuclei isolation from human postmortem brain tissue. J Vis Exp. 2008. 10.3791/914 PMC3233860 19078943

[66] Kadkhodaei B, Ito T, Joodmardi E, Mattsson B, Rouillard C, Carta M, Muramatsu S, Sumi-Ichinose C, Nomura T, Metzger D (2009). Nurr1 is required for maintenance of maturing and adult midbrain dopamine neurons. J Neurosci.

[67] Bannon MJ, Pruetz B, Manning-Bog AB, Whitty CJ, Michelhaugh SK, Sacchetti P, Granneman JG, Mash DC, Schmidt CJ (2002). Decreased expression of the transcription factor NURR1 in dopamine neurons of cocaine abusers. Proc Natl Acad Sci U S A.

[68] Chu Y, Le W, Kompoliti K, Jankovic J, Mufson EJ, Kordower JH (2006). Nurr1 in Parkinson’s disease and related disorders. J Comp Neurol.

[69] Bryois J, Skene NG, Hansen TF, Kogelman L, Watson HJ, Brueggeman L, Breen G, Bulik CM, Arenas E, Hjerling-Leffler J, Sullivan PF. Genetic identification of cell types underlying brain complex traits yields novel insights into the etiology of Parkinson’s disease. bioRxiv. 2019:528463.

[70] Horwitz T, Lam K, Chen Y, Xia Y, Liu C (2019). A decade in psychiatric GWAS research. Mol Psychiatry.

[71] Cremer T, Cremer M (2010). Chromosome territories. Cold Spring Harb Perspect Biol.

[72] Dixon JR, Gorkin DU, Ren B (2016). Chromatin domains: the unit of chromosome organization. Mol Cell.

[73] Stark AK, Pakkenberg B (2004). Histological changes of the dopaminergic nigrostriatal system in aging. Cell Tissue Res.

[74] Adey A, Morrison HG, Asan XX, Kitzman JO, Turner EH, Stackhouse B, AP MK, Caruccio NC, Zhang X, Shendure J (2010). Rapid, low-input, low-bias construction of shotgun fragment libraries by high-density in vitro transposition. Genome Biol.

[75] Szklarczyk D, Morris JH, Cook H, Kuhn M, Wyder S, Simonovic M, Santos A, Doncheva NT, Roth A, Bork P (2017). The STRING database in 2017: quality-controlled protein-protein association networks, made broadly accessible. Nucleic Acids Res.

[76] Ay M, Jin H, Harischandra DS, Asaithambi A, Kanthasamy A, Anantharam V, Kanthasamy AG (2015). Molecular cloning, epigenetic regulation, and functional characterization of Prkd1 gene promoter in dopaminergic cell culture models of Parkinson’s disease. J Neurochem.

[77] Hwu WL, Lee NC, Chien YH, Muramatsu S, Ichinose H (2013). AADC deficiency: occurring in humans, modeled in rodents. Adv Pharmacol.

[78] Ahuja R, Pinyol R, Reichenbach N, Custer L, Klingensmith J, Kessels MM, Qualmann B (2007). Cordon-bleu is an actin nucleation factor and controls neuronal morphology. Cell.

[79] Paulsen J, Sekelja M, Oldenburg AR, Barateau A, Briand N, Delbarre E, Shah A, Sorensen AL, Vigouroux C, Buendia B, Collas P (2017). Chrom3D: three-dimensional genome modeling from Hi-C and nuclear lamin-genome contacts. Genome Biol.

[80] Cordeira JW, Frank L, Sena-Esteves M, Pothos EN, Rios M (2010). Brain-derived neurotrophic factor regulates hedonic feeding by acting on the mesolimbic dopamine system. J Neurosci.

[81] Wang ZJ, Martin JA, Mueller LE, Caccamise A, Werner CT, Neve RL, Gancarz AM, Li JX, Dietz DM (2016). BRG1 in the nucleus accumbens regulates cocaine-seeking behavior. Biol Psychiatry.

[82] Gancarz AM, Wang ZJ, Schroeder GL, Damez-Werno D, Braunscheidel KM, Mueller LE, Humby MS, Caccamise A, Martin JA, Dietz KC (2015). Activin receptor signaling regulates cocaine-primed behavioral and morphological plasticity. Nat Neurosci.

[83] Zhang J, Pho V, Bonasera SJ, Holtzman J, Tang AT, Hellmuth J, Tang S, Janak PH, Tecott LH, Huang EJ (2007). Essential function of HIPK2 in TGFbeta-dependent survival of midbrain dopamine neurons. Nat Neurosci.

[84] Gorkin DU, Leung D, Ren B (2014). The 3D genome in transcriptional regulation and pluripotency. Cell Stem Cell.

[85] Mele M, Rinn JL (2016). “Cat’s Cradling” the 3D genome by the act of LncRNA transcription. Mol Cell.

[86] Hubner MR, Eckersley-Maslin MA, Spector DL (2013). Chromatin organization and transcriptional regulation. Curr Opin Genet Dev.

[87] Loviglio MN, Leleu M, Mannik K, Passeggeri M, Giannuzzi G, van der Werf I, Waszak SM, Zazhytska M, Roberts-Caldeira I, Gheldof N (2017). Chromosomal contacts connect loci associated with autism, BMI and head circumference phenotypes. Mol Psychiatry.

[88] Trost S, Diekhof EK, Mohr H, Vieker H, Kramer B, Wolf C, Keil M, Dechent P, Binder EB, Gruber O (2016). Investigating the impact of a genome-wide supported bipolar risk variant of MAD1L1 on the human reward system. Neuropsychopharmacology.

[89] Rampino A, Marakhovskaia A, Soares-Silva T, Torretta S, Veneziani F, Beaulieu JM (2018). Antipsychotic drug responsiveness and dopamine receptor signaling; old players and new prospects. Front Psychiatry.

[90] Lohmann K, Masuho I, Patil DN, Baumann H, Hebert E, Steinrucke S, Trujillano D, Skamangas NK, Dobricic V, Huning I (2017). Novel GNB1 mutations disrupt assembly and function of G protein heterotrimers and cause global developmental delay in humans. Hum Mol Genet.

[91] Le Hellard S, Theisen FM, Haberhausen M, Raeder MB, Ferno J, Gebhardt S, Hinney A, Remschmidt H, Krieg JC, Mehler-Wex C (2009). Association between the insulin-induced gene 2 (INSIG2) and weight gain in a German sample of antipsychotic-treated schizophrenic patients: perturbation of SREBP-controlled lipogenesis in drug-related metabolic adverse effects?. Mol Psychiatry.

[92] Bauer S, Wanninger J, Schmidhofer S, Weigert J, Neumeier M, Dorn C, Hellerbrand C, Zimara N, Schaffler A, Aslanidis C, Buechler C (2011). Sterol regulatory element-binding protein 2 (SREBP2) activation after excess triglyceride storage induces chemerin in hypertrophic adipocytes. Endocrinology.

[93] Kim DH, Sabatini DM (2004). Raptor and mTOR: subunits of a nutrient-sensitive complex. Curr Top Microbiol Immunol.

[94] Loviglio MN, Arbogast T, Jonch AE, Collins SC, Popadin K, Bonnet CS, Giannuzzi G, Maillard AM, Jacquemont S (2017). The immune signaling adaptor LAT contributes to the neuroanatomical phenotype of 16p11.2 BP2-BP3 CNVs. Am J Hum Genet.

[95] Chang CC, Chow CC, Tellier LC, Vattikuti S, Purcell SM, Lee JJ (2015). Second-generation PLINK: rising to the challenge of larger and richer datasets. Gigascience.

[96] Cave JW, Baker H (2009). Dopamine systems in the forebrain. Adv Exp Med Biol.

[97] Vantaggiato C, Panzeri E, Citterio A, Orso G, Pozzi M (2019). Antipsychotics promote metabolic disorders disrupting cellular lipid metabolism and trafficking. Trends Endocrinol Metab.

[98] Enez Darcin A, Yalcin Cavus S, Dilbaz N, Kaya H, Dogan E (2015). Metabolic syndrome in drug-naive and drug-free patients with schizophrenia and in their siblings. Schizophr Res.

[99] Wu C, Garamszegi SP, Xie X, Mash DC (2017). Altered dopamine synaptic markers in postmortem brain of obese subjects. Front Hum Neurosci.

[100] Sun X, Luquet S, Small DM (2017). DRD2: bridging the genome and Ingestive behavior. Trends Cogn Sci.

[101] Eberle D, Hegarty B, Bossard P, Ferre P, Foufelle F (2004). SREBP transcription factors: master regulators of lipid homeostasis. Biochimie.

[102] Ziegler AB, Thiele C, Tenedini F, Richard M, Leyendecker P, Hoermann A, Soba P, Tavosanis G (2017). Cell-autonomous control of neuronal dendrite expansion via the fatty acid synthesis regulator SREBP. Cell Rep.

[103] Bosia M, Buonocore M, Bechi M, Santarelli L, Spangaro M, Cocchi F, Guglielmino C, Bianchi L, Bringheli S, Bosinelli F, Cavallaro R (2018). Improving cognition to increase treatment efficacy in schizophrenia: effects of metabolic syndrome on cognitive Remediation’s outcome. Front Psychiatry.

[104] Wang H, Xu X, Nguyen CM, Liu Y, Gao Y, Lin X, Daley T, Kipniss NH, La Russa M, Qi LS (2018). CRISPR-mediated programmable 3D genome positioning and nuclear organization. Cell.

[105] Ma W, Ay F, Lee C, Gulsoy G, Deng X, Cook S, Hesson J, Cavanaugh C, Ware CB, Krumm A (2015). Fine-scale chromatin interaction maps reveal the cis-regulatory landscape of human lincRNA genes. Nat Methods.

[106] Nagano T, Lubling Y, Varnai C, Dudley C, Leung W, Baran Y, Mendelson Cohen N, Wingett S, Fraser P, Tanay A (2017). Cell-cycle dynamics of chromosomal organization at single-cell resolution. Nature.

[107] Wang S, Su JH, Beliveau BJ, Bintu B, Moffitt JR, Wu CT, Zhuang X (2016). Spatial organization of chromatin domains and compartments in single chromosomes. Science.

[108] Fudenberg G, Imakaev M (2017). FISH-ing for captured contacts: towards reconciling FISH and 3C. Nat Methods.

[109] Finucane HK, Reshef YA, Anttila V, Slowikowski K, Gusev A, Byrnes A, Gazal S, Loh PR, Lareau C, Shoresh N (2018). Heritability enrichment of specifically expressed genes identifies disease-relevant tissues and cell types. Nat Genet.

[110] Espeso-Gil S, et al. “A chromosomal connectome for psychiatric and metabolic risk variants in adult dopaminergic neurons”. Genome Medicine. HiC and midbrain RNAseq. syn20833047, URL: https://www.synapse.org/#!Synapse:syn20833047 , registration required; access date for registered users 10/02/2019. 10.1186/s13073-020-0715-x PMC7031924 32075678

[111] Espeso-Gil S, et al. A chromosomal connectome for psychiatric and metabolic risk variants in adult dopaminergic neurons”. Genome Medicine PFC/ACC RNAseq. syn20545534 , https://www.synapse.org/#!Synapse:syn20545534, registration required. Access date for registered users: 04/24/2019. RNAseq browser UCSC visualization: https://genome.ucsc.edu/s/sespeso/EspesoGil_Halene2019 10.1186/s13073-020-0715-x PMC7031924 32075678

[112] Rajarajan P, et al. “Neuron-specific signatures in the chromosomal connectome associated with schizophrenia risk”. Science. Datasets used in the present study: HiC from NPCs, Astrocytes and Ngn2 (syn12979101, https://www.synapse.org/#!Synapse:syn12979101 , registration required. Access date for registered users: 04/15/2019). 10.1126/science.aat4311 PMC6408958 30545851

[113] Rao SSP, et al. “A 3D map of the human genome at kilobase resolution reveals principles of chromatin looping”. Cell. HiC datasets from GM12978, IMR90, KBM7 and HUVEC (GSE63525, https://www.ncbi.nlm.nih.gov/geo/query/acc.cgi?acc=GSE63525. Public access date 12/11/1014). 10.1016/j.cell.2014.11.021 PMC5635824 25497547

[114] Won H, et al. “Chromosome conformation elucidates regulatory relationships in developing human brain”. Nature. Datasets used in the present study: HiC from fetal cortical plate and germinal zone. GSE63525 https://www.ncbi.nlm.nih.gov/geo/query/acc.cgi?acc=GSE63525 and phs001190.v1.p1 https://www.ncbi.nlm.nih.gov/projects/gap/cgi-bin/study.cgi?study_id=phs001190.v1.p1) Access date 10/27/2016. 10.1038/nature19847 PMC5358922 27760116

