# Supplementary material for: A chromosomal connectome for psychiatric and metabolic risk variants in adult dopaminergic neurons
Source: Genome Med. 2020 Feb 19;12:19. doi: 10.1186/s13073-020-0715-x (PMC7031924; doi:10.1186/s13073-020-0715-x)
Supplement: Supplementary file 1 — Additional file 1. Supplemental Methods. [file 13073_2020_715_MOESM1_ESM.docx]

**Tn5 Hi-C Protocol***; (ESPESO-Gil, Halene ET AL*)

**Nuclei isolation and fixation from mouse and human brain tissue**

1. Start with fresh or fresh frozen brain tissue. Tissue can be saved at -80°C until the day of use.
2. Dounce brain tissue in 4ml of nuclei lysis buffer, and then transfer into a new 15ml tube (352099, FALCON)
3. Add fresh 108μl of 37% of formaldehyde, and rotate for 5min at room temperature
4. Add 300μl of 2M glycine, rotate for 10 min at room temperature
5. Centrifuge at 4000rpm, 5min, 4°C
6. Remove supernatant and resuspend pellet in 5ml of lysis buffer, and then mix with 5ml of sucrose buffer
7. Centrifuge at 4000rpm, 5min, 4°C
8. Remove supernatant, resuspend pellet in 5 ml of lysis buffer, and then go through the 70μm cell strainer
9. Transfer the filtered nuclei solution into an ultracentrifuge tube and load 9 ml of sucrose solution to the bottom of nuclei solution
10. Centrifuge at 24,000rpm, 1hr, 4°C (Thermo Scientific SureSpin 630 (17 mL) Rotor, the g force is about 100,000 x g)
11. Clean supernatant carefully, continue with FACS or save the nuclei pellet at -80°C for later use

**FACS**

1. Prepare collection tubes by coating with 10% BSA for at least 30 min at RT
2. Take out nuclei pellet, thaw on ice, resuspend in 500 ul of DPBS
3. Make a mixture of 500 ul of DPBS + 10 μl of 10%BSA + 0.5 μl of anti-NeuN-488 (EMD MILLIPORE CORP MAB377X). anti-Nurr1 antibody (E-20) (Santa Cruz, sc-990) was use for dopaminergic nuclei sorting from human postmortem brain from ventral midbrain/ substantia nigra.
4. Mix the 500μl of antibody mixture to 500μl of nuclei solution
5. Incubate at 4°C for at least 45 min
6. After incubation, add 0.5μl of DAPI and FACS to collect NeuN+ and NeuN- nuclei
7. After sorting, pellet nuclei at 4000rpm, 5min, 4°C
8. Carefully remove supernatant and nuclei pellet can be saved at -80°C for future use

**MboI Digest**

1. Take out sorted nuclei pellet from -80°C freezer
2. Resuspend pellet in 50μl of 0.5% sodium dodecyl sulfate (SDS) and incubate at 65°C for 5 minutes.
3. Add 145μl of water and 25μl of 10% Triton X-100 (Sigma, 93443) to quench the SDS, and incubate at 37°C for 15 minutes.
4. Add 25μl of 10X NEBuffer2 and 20μl (100U) of MboI restriction enzyme (NEB, R0147) and digest chromatin  overnight at 37°C with rotation.

**T4 Ligation**

1. Incubate at 65°C for 20 minutes to inactivate MboI, then cool to room temperature.
2. For each sample, add: 
   698μl of water 
   120μl of 10X NEB T4 DNA ligase buffer (NEB, B0202)
   100μl of 10% Triton X-100 
   12μl of 10mg/ml Bovine Serum Albumin (100X BSA)
   5μl of 400U/μl T4 DNA Ligase (NEB, M0202)
   (Optional_QC: for No Ligase control, replace T4 ligase with water)
3. Incubate at room temperature for 4 hours with slow rotation
4. Centrifuge at 10,000rpm for 10 minutes, carefully remove supernatant

**Tn5 transposes treatment**

1. For each sample, add (Illumina, Cat#FC-121-1030):  
   20μl of water 
   25μl 2x of Tagment DNA Buffer
   5ul of Tagment DNA Enzyme
2. Incubate at 37°C for 30 minutes

**PKK digestion and reverse crosslinking**

11. Add 450μl of PK lysis buffer and 10μl proteinase K (10mg/ml) (Fisher, Cat# 25530015), and incubate at 55°C for 30 minutes

12. Incubate at 65°C overnight

**DNA precipitation**

13. For each tube, add:

2μl GlycoBlue (Ambion, Cat# AM9515)

50μl of 3M sodium acetate, pH 5.2

1400μl of pre-chilled ethanol

14. Invert mix well and incubate at -80°C for 1 hr

15. Centrifuge at max speed, 4°C for 30 minutes

16. Discard the supernatant and add in 800μl of 70% ethanol.

17. Centrifuge at max speed, 4°C for 15 minutes

18. Remove all supernatant, air dry the pellet

19. Dissolve the pellet in 20μl of EB buffer

**Library preparation**

20.For PCR reaction (Illumina, Cat#FC-121-1030), add:

20μl of DNA sample

25μl of PCR master mix

2.5μl Index 5

2.5μl Index 7

21. PCR amplification

72°C for 3 minutes

98°C for 30 seconds

8 or 12 cycles of:

-98°C for 10 seconds

-63°C for 30 seconds

-72°C for 3 minutes

Hold at 10°C

(Take 1μl and check fragment size on Bioanalyzer)

**Ampure beads size selection**

22. Add 30 ul of Ampure beads (Beckman Coulter, Cat# B23318), mix and incubate at room temperature for 5 minutes

23. Collect beads with magnet and wash twice with fresh 80% ethanol

24. Air dry beads

25. Resuspend beads in 20 ul of EB buffer (Qiagen) (library size, 150-500bp)

26. Transfer supernatant from step 23 to a new tube and mix with 60 ul of Ampure beads, incubate at room temperature for 5 minutes

27. Collect beads with magnet and wash twice with fresh 80% ethanol

28. Air dry beads

29. Resuspend beads in 20 ul of EB buffer (library size, 800-1200bp)

30. Measure concentration by Qubit and check fragment size on Bioanalyzer.

31. Save libraries (two size fragments) at -80°C

**Reagents and Materials:**

**Nuclei lysis buffer**

0.32M Sucrose 5.47 g

5 mM CaCl2 250 ul

3 mM Mg(Ace)2 150 ul

0.1 mM EDTA 10 ul

10mM Tris-HCl, pH8 500 ul

1 mM DTT 17 ul

0.1% Triton X-100 50 ul

--- Adjust with ddH2O to 50 mL

**Sucrose Solution**

1.8 M Sucrose 30.78 g

3 mM Mg(Ace)2 150 ul

1 mM DTT 17 ul

10 mM Tris-HCl, pH8 500 ul

--- Adjust with ddH2O to 50 mL

**PK lysis buffer**

100mM Trix, pH 8

200mM NaCl

5 mM edta

0.1%SDS
