# Supplementary material for: A chromosomal connectome for psychiatric and metabolic risk variants in adult dopaminergic neurons
Source: Genome Med. 2020 Feb 19;12:19. doi: 10.1186/s13073-020-0715-x (PMC7031924; doi:10.1186/s13073-020-0715-x)
Supplement: Supplementary file 3 — Additional file 3. Supplementary Figures. [file 13073_2020_715_MOESM3_ESM.pdf]

Figure S1

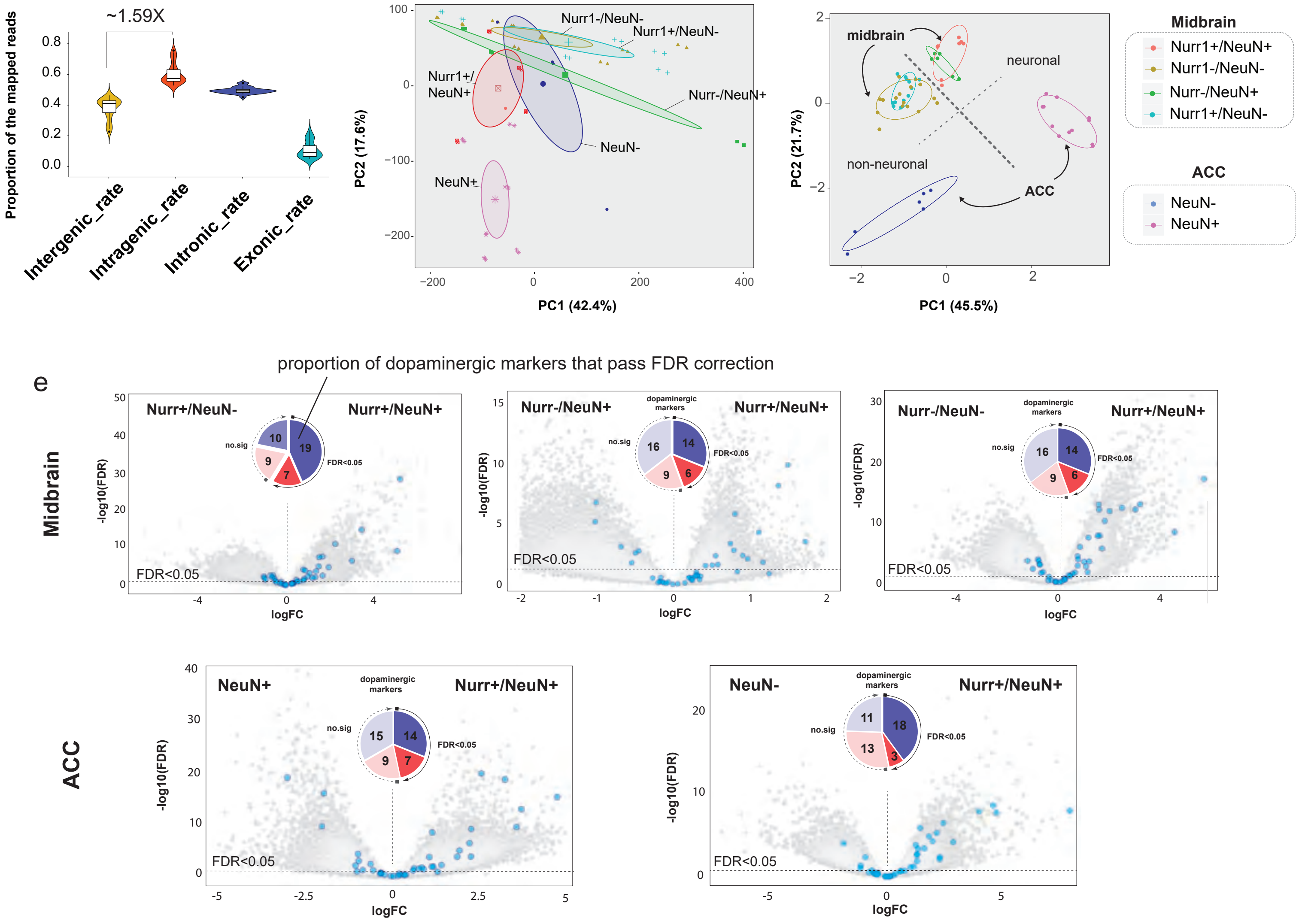
a
~1.59X
Proportion of the mapped reads
Intergenic_rate
Intragenic_rate
Intronic_rate
Exonic_rate
b
Genome-wide principal component analysis (PCA)
Nurr1-/NeuN-
Nurr1+/NeuN-
Nurr1+/NeuN+
Nurr-/NeuN+
NeuN-
NeuN+
PC1 (42.4%)
PC2 (17.6%)
c
Dopaminergic expression PCA
midbrain
neuronal
non-neuronal
ACC
PC1 (45.5%)
PC2 (21.7%)
Midbrain
Nurr1+/NeuN+
Nurr1-/NeuN-
Nurr-/NeuN+
Nurr1+/NeuN-
ACC
NeuN-
NeuN+
e
proportion of dopaminergic markers that pass FDR correction
Midbrain
Nurr+/NeuN-
Nurr+/NeuN+
Nurr-/NeuN+
Nurr-/NeuN-
dopaminergic markers
no.sig
FDR<0.05
-log10(FDR)
logFC
ACC
NeuN+
NeuN-

***Supplementary Figure 1. Transcriptomic analysis of sorted nuclei from anterior cingulate cortex (ACC) and the ventral midbrain*.** **a)** Genomic features of mapped reads from nucRNAseq datasets (*Additional File 2: Table S2*) using RNA-SeQC[39]. Violin plots show proportions of intergenic, intragenic, intronic and exonic fractions for the N=30 cell type specific nucRNAseq libraries (*Additional File: Table S2*). **b)** Genome-wide PCA analysis of nucRNAseq from six sorted nuclei populations (4 midbrain, 2 cortical nuclei populations) as indicated. **c)** PCA analyses of 45 transcripts selected from mouse midbrain single cell RNAseq resource[41] for the six sorted nuclei populations as indicated**. d)** Differential nucRNA-seq analysis of five different nuclei populations from midbrain and cortex, each compared against midbrain dopaminergic neuron $Nurr1^+/NeuN^+$ using edgeR[110] wrapper tool RUVseq[40] . Pie charts denote the proportion of dopaminergic markers that pass the FDR cutoff and the direction of the fold change (blue denotes upregulated and red downregulated in $Nurr1^+/NeuN^+$ population).

Figure S2

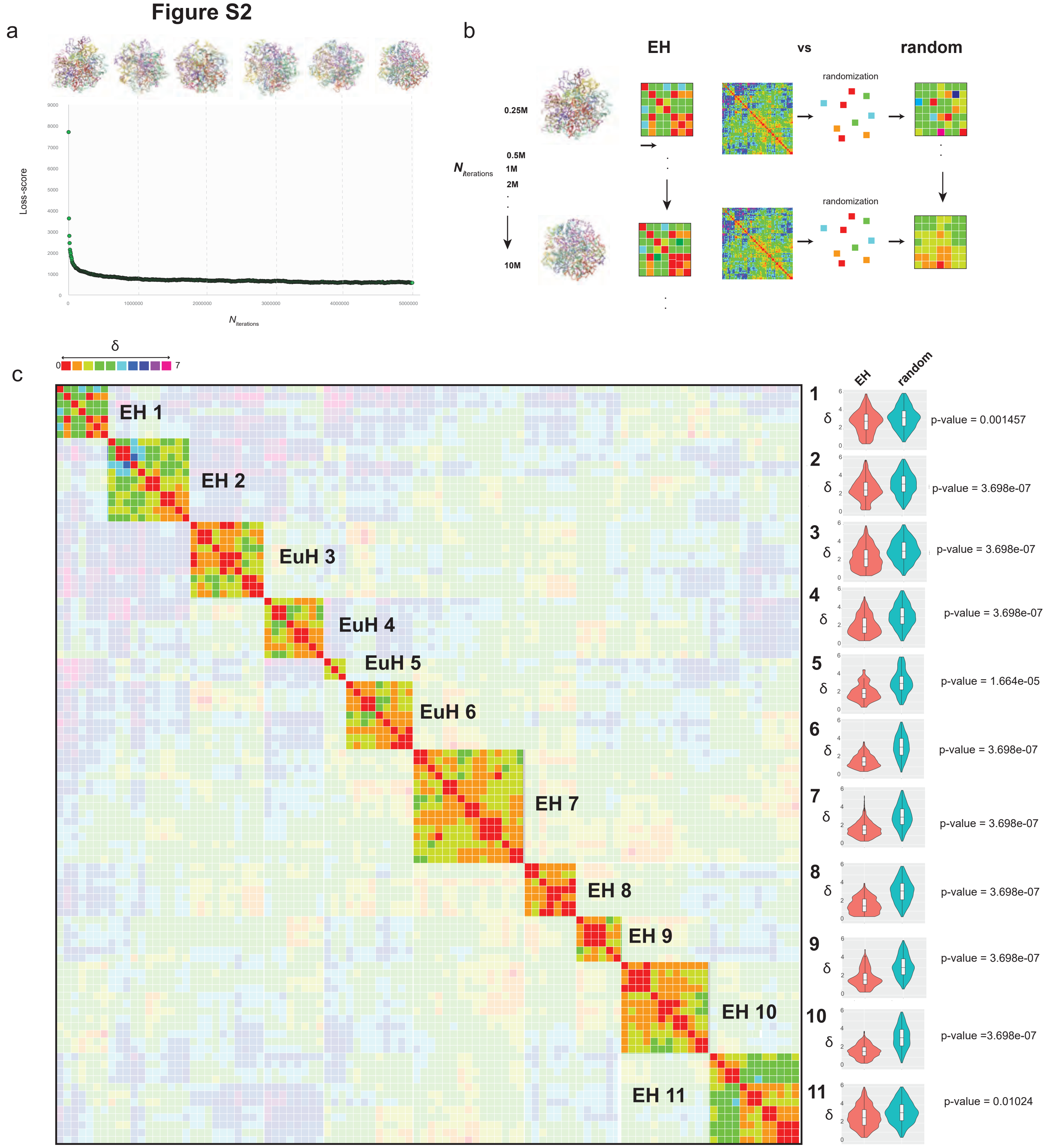
a
Loss-score
$N_{iterations}$
b
EH
vs
random
0.25M
0.5M
1M
2M
10M
$N_{iterations}$
randomization
randomization
c
δ
0
7
EH 1
EH 2
EuH 3
EuH 4
EuH 5
EuH 6
EH 7
EH 8
EH 9
EH 10
EH 11
EH
random
1 p-value = 0.001457
2 p-value = 3.698e-07
3 p-value = 3.698e-07
4 p-value = 3.698e-07
5 p-value = 1.664e-05
6 p-value = 3.698e-07
7 p-value = 3.698e-07
8 p-value = 3.698e-07
9 p-value = 3.698e-07
10 p-value =3.698e-07
11 p-value = 0.01024

Supplementary Figure 2. *In silico* Nurr1⁺/NeuN⁺ chrom3D model stability across multiple iterations.

Benchmark analysis for the *in-silico* chrom3D model stability across multiple iterations **a**) genome-wide and (**b,c**) by Euclidean hotspot (b,c). **a)** Loss score plot measuring conformation sparsity of the *in-silico* model with successive increase in iterations (0.25M to 5M) across different runs of chrom3D. Note that the model starts to become stable after 1M of iterations. **b, c)** In order to assess the statistical confidence for each EH to be found with the exact same pairwise distance cluster between its domains, chrom3D was run 12 times with multiple iterations (from 250k, 500k, 1M, to 10M of iterations) and EH pair-wise distances were calculated along with random domains pair-wise distances in the model. As the distribution of the distances do not follow a Normal distribution (tested by a Shapiro and Andersen normality test), a non-paired Wilcox test was used to estimate the significance to find the same structure against randomness across all chrom3D iterations.

Figure S3

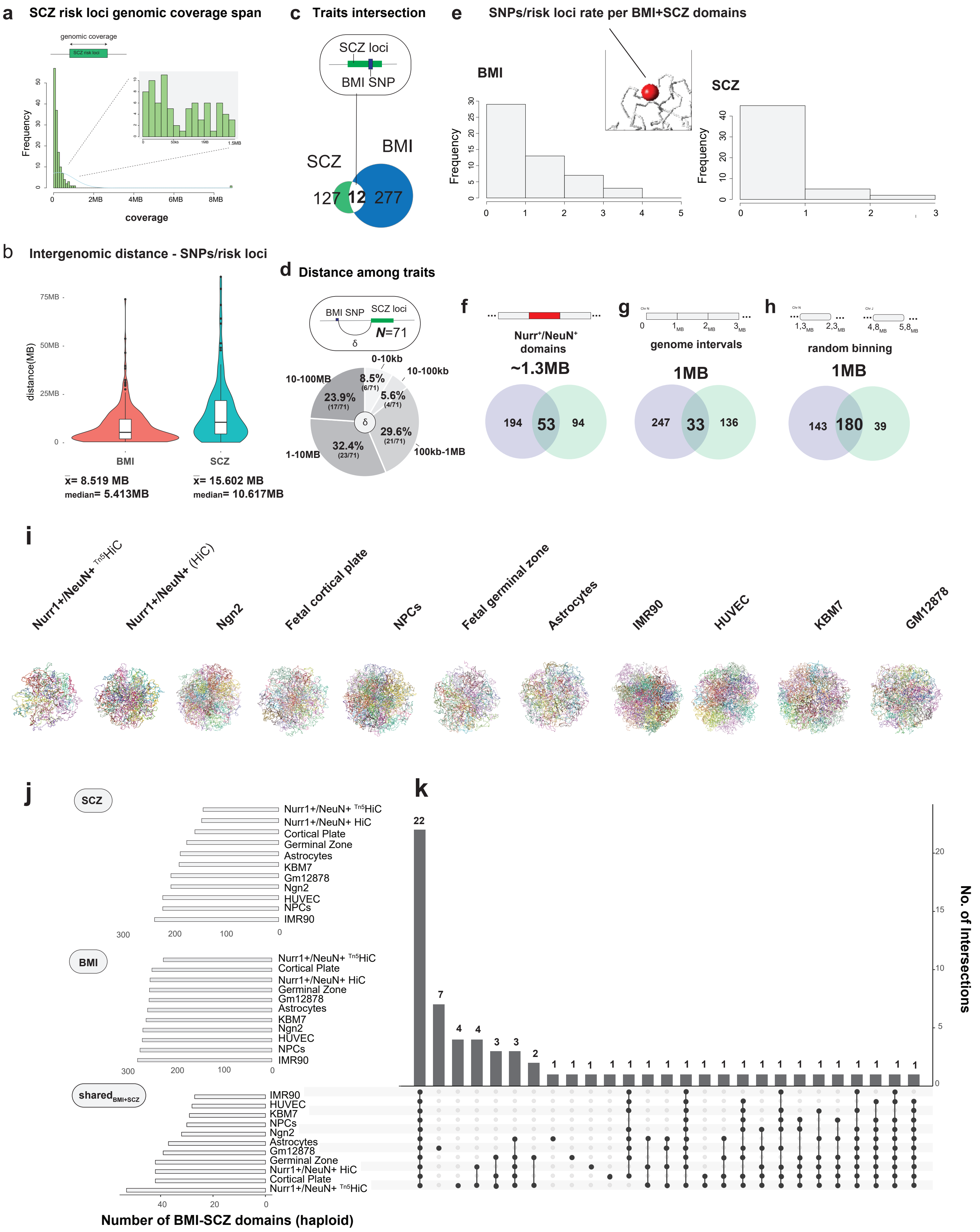

a SCZ risk loci genomic coverage span
genomic coverage
SCZ risk loci
Frequency
coverage
0
2MB
4MB
6MB
8MB
c Traits intersection
SCZ loci
BMI SNP
SCZ
BMI
127
12
277
e SNPs/risk loci rate per BMI+SCZ domains
BMI
SCZ
Frequency
b Intergenomic distance - SNPs/risk loci
distance(MB)
75MB
50MB
25MB
0
BMI
SCZ
x̄= 8.519 MB
median= 5.413MB
x̄= 15.602 MB
median= 10.617MB
d Distance among traits
BMI SNP
SCZ loci
N=71
δ
0-10kb
10-100kb
10-100MB
8.5% (6/71)
5.6% (4/71)
23.9% (17/71)
29.6% (21/71)
32.4% (23/71)
1-10MB
100kb-1MB
f
Nurr+/NeuN+ domains
~1.3MB
194
53
94
g
genome intervals
1MB
247
33
136
h
random binning
1MB
143
180
39
i
Nurr1+/NeuN+ Tn5HiC
Nurr1+/NeuN+ (HiC)
Ngn2
Fetal cortical plate
NPCs
Fetal germinal zone
Astrocytes
IMR90
HUVEC
KBM7
GM12878
j
SCZ
Nurr1+/NeuN+ Tn5HiC
Nurr1+/NeuN+ HiC
Cortical Plate
Germinal Zone
Astrocytes
KBM7
Gm12878
Ngn2
HUVEC
NPCs
IMR90
BMI
Nurr1+/NeuN+ Tn5HiC
Cortical Plate
Nurr1+/NeuN+ HiC
Germinal Zone
Gm12878
Astrocytes
KBM7
Ngn2
HUVEC
NPCs
IMR90
shared BMI+SCZ
IMR90
HUVEC
KBM7
NPCs
Ngn2
Astrocytes
Gm12878
Germinal Zone
Nurr1+/NeuN+ HiC
Cortical Plate
Nurr1+/NeuN+ Tn5HiC
Number of BMI-SCZ domains (haploid)
k
22
7
4
4
3
3
2
No. of Intersections

Supplementary Figure 3 Linear and 3D conformation characterization of BMI and SCZ.

a) Histogram of schizophrenia genomic coverage. SCZ loci average in length ~ 256kb$\pm$749. **b**) Genomic distance between BMI SNPs (left) and schizophrenia risk loci (right) **c**) BMI SNPs and schizophrenia loci genomic Venn plot intersection showing that only 12 BMI SNPs fall into schizophrenia risk loci. **d**) 2D linear genome exploration of contiguous BMI and schizophrenia polymorphisms (N=71 SCZ risk loci located next to BMI risk SNP or vice versa). Pie chart showing the distance interval percentage among BMI SNPs and schizophrenia risk loci (interval hits versus total number showed in parenthesis). **e**) Histogram of BMI SNPs (left) and schizophrenia risk loci (right) falling into the same Nurr1+/NeuN+ 3D model domain. **f**) Intersection of both BMI and schizophrenia traits with Nurr1^{+}/NeuN^{+} 3D model domains. **g**) genomic intervals observed at 1Mb and **h**) computed by random binning of 1Mb. **i**) *In silico* 3D cell models from Hi-C datasets generated from various cell types and tissues as indicated, at 50kb and 1MB resolution of intra-chromosomal and inter-chromosomal interactions respectively with 5M Chrom3D iterations (see Methods). **j**) Intersection of (top) schizophrenia risk) loci, (middle) BMI SNPs and (bottom) co-localization of both traits in 3D domains of all cell types present in the analysis. k) Number of BMI-SCZ domains shared across all 3D models.

Figure S4

BMI-SCZ$_{domains}$ vs other traits

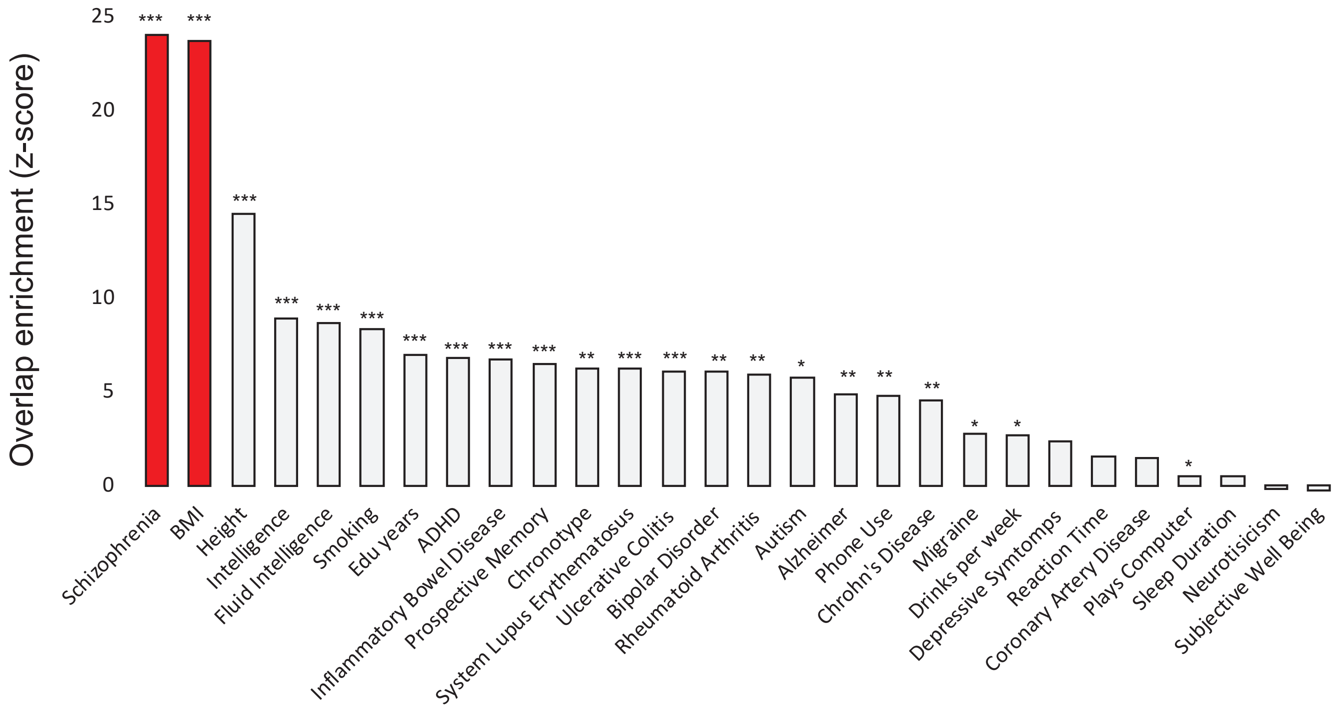

Supplementary Figure 4: Permutation analysis of BMI-SCZ shared domains versus 28 GWAS traits. Bar plot showing the strength of the association of BMI-SCZ shared domains over 28 traits (selection of significant SNPs at p value < $5*10^{-8}$) . The overlap enrichment is measured by z-scores that were calculated permuting BMI-SCZ domains 10k times randomly over all domains called in Nurr1^{+}/NeuN^{+} nuclei. Values represent the distance between the expected and the observed value, measured in standard deviations. Note that BMI and SCZ are the two top ranking GWAS; other traits were also significantly enriched (asterisks, *** pvalue <0.001 , ** pvalue < 0.01, * pvalue < 0.05) but the strength of the associations are considerable less when compared to BMI and SCZ each separately (red bars). The following GWAS summary statistics were used in this analyses (PMID numbers): Type 2 Diabetes (30297969); Drinks per week and Ever smoker(30643258); Prospective memory, Fluid intelligence score, Migraine, Phone use, Gaming defined as Plays computer (31427789); Reaction time (29844566); Height (25282103); Coronary artery disease (26343387); Crohn's disease, Ulcerative Colitis; Inflammatory Bowel disease (26192919); Rheumatoid Arthritis (24390342); Systemic Lupus Erythematosus (26502338); BMI (25673413); Chronotype and Sleep duration (27494321); Insomnia (28604731); Depressive Symptoms, Neuroticism, Subjective Well Being (27089181); Intelligence (28530673); Education Years (28197077); Attention Deficit/ Hyperactivity Disorder=ADHD (30478444); Alzheimer's Disease (24162737); Autism (30804558); Depression (); Bipolar Disorder (31043756); SCZ (25056061).

Figure S5

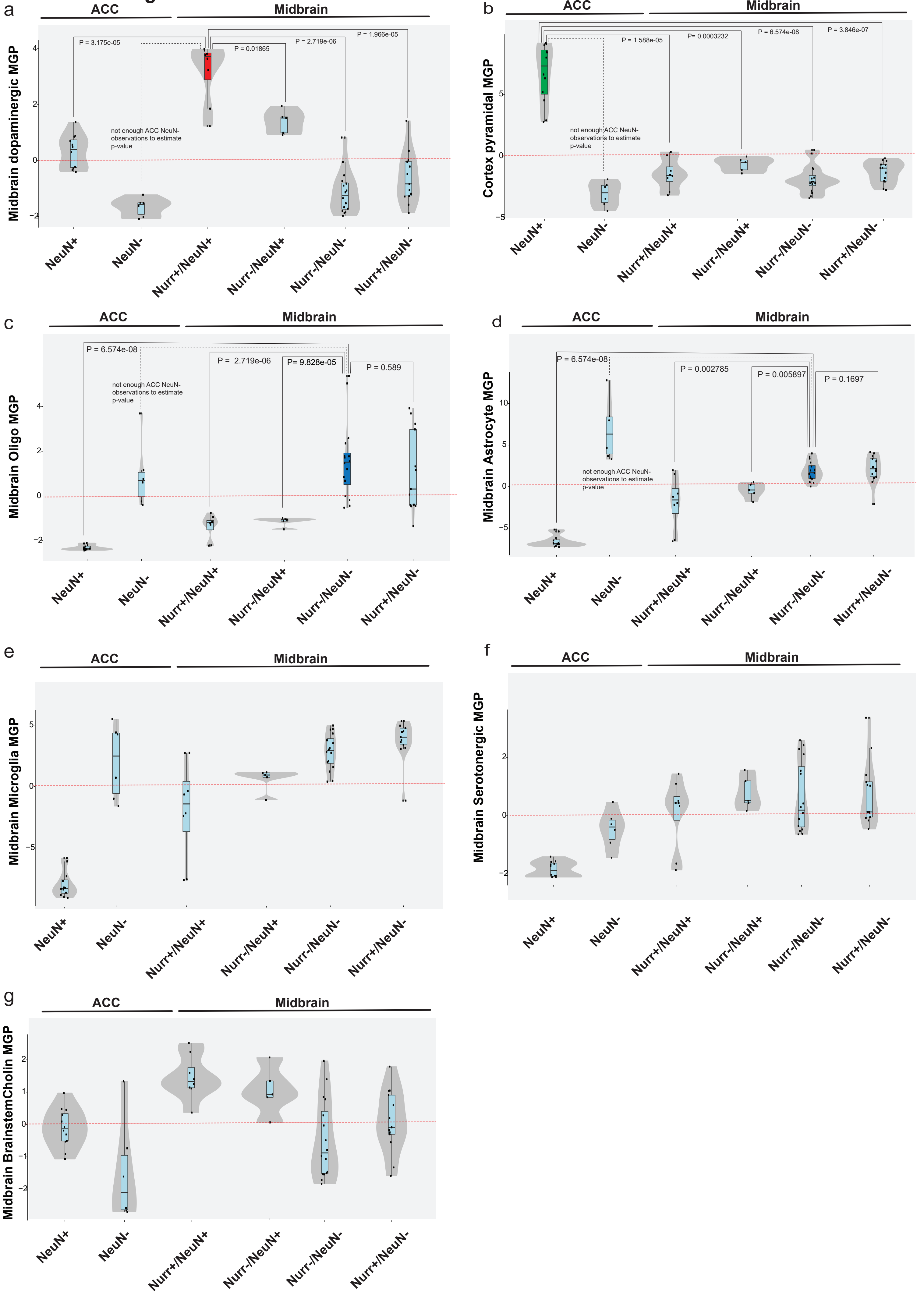

a
ACC
Midbrain
Midbrain dopaminergic MGP
P = 3.175e-05
P = 0.01865
P = 2.719e-06
P = 1.966e-05
not enough ACC NeuN- observations to estimate p-value
NeuN+
NeuN-
Nurr+/NeuN+
Nurr-/NeuN+
Nurr-/NeuN-
Nurr+/NeuN-
b
ACC
Midbrain
Cortex pyramidal MGP
P = 1.588e-05
P= 0.0003232
P = 6.574e-08
P = 3.846e-07
not enough ACC NeuN- observations to estimate p-value
c
ACC
Midbrain
Midbrain Oligo MGP
P = 6.574e-08
P = 2.719e-06
P= 9.828e-05
P = 0.589
not enough ACC NeuN- observations to estimate p-value
d
ACC
Midbrain
Midbrain Astrocyte MGP
P = 6.574e-08
P = 0.002785
P = 0.005897
P = 0.1697
not enough ACC NeuN- observations to estimate p-value
e
ACC
Midbrain
Midbrain Microglia MGP
f
ACC
Midbrain
Midbrain Serotonergic MGP
g
ACC
Midbrain
Midbrain BrainstemCholin MGP

Supplementary Figure 5. Cell-specific marker gene expression. Gene expression by cell type, using single-cell curated data from *markerGeneProfile* utility in neuroexpresso[42] . The four nuclei subpopulations from ventral midbrain (Nurr1$^+$/NeuN$^+$, Nurr1$^-$/NeuN$^+$, Nurr1$^+$/NeuN$^-$, Nurr1$^-$/ NeuN$^{-)}$ and two cortical (NeuN$^+$ and NeuN$^-$) subpopulations of nuclei of the present study were compared for marker gene expression representing seven cell types including brainstem dopaminergic and cholinergic and serotonergic neurons, microglia and oligodendrocytes and astrocytes, and cortical pyramidal neurons, as indicated on Y-axes. **a)** Violin plots show significantly increased expression of dopaminergic marker genes in Nurr1$^+$/NeuN+ MDN nuclei populations compared to each of the remaining five non-dopaminergic nuclei populations (Wilcoxon p). **b)** cortical pyramidal neuron marker expression is highest in cortex ACC NeuN$^+$. **c)** oligodendrocyte, **d)** astrocyte, **e)** microglia, **f)** serotonergic and **g**) cholinergic marker gene expression.

Figure S6

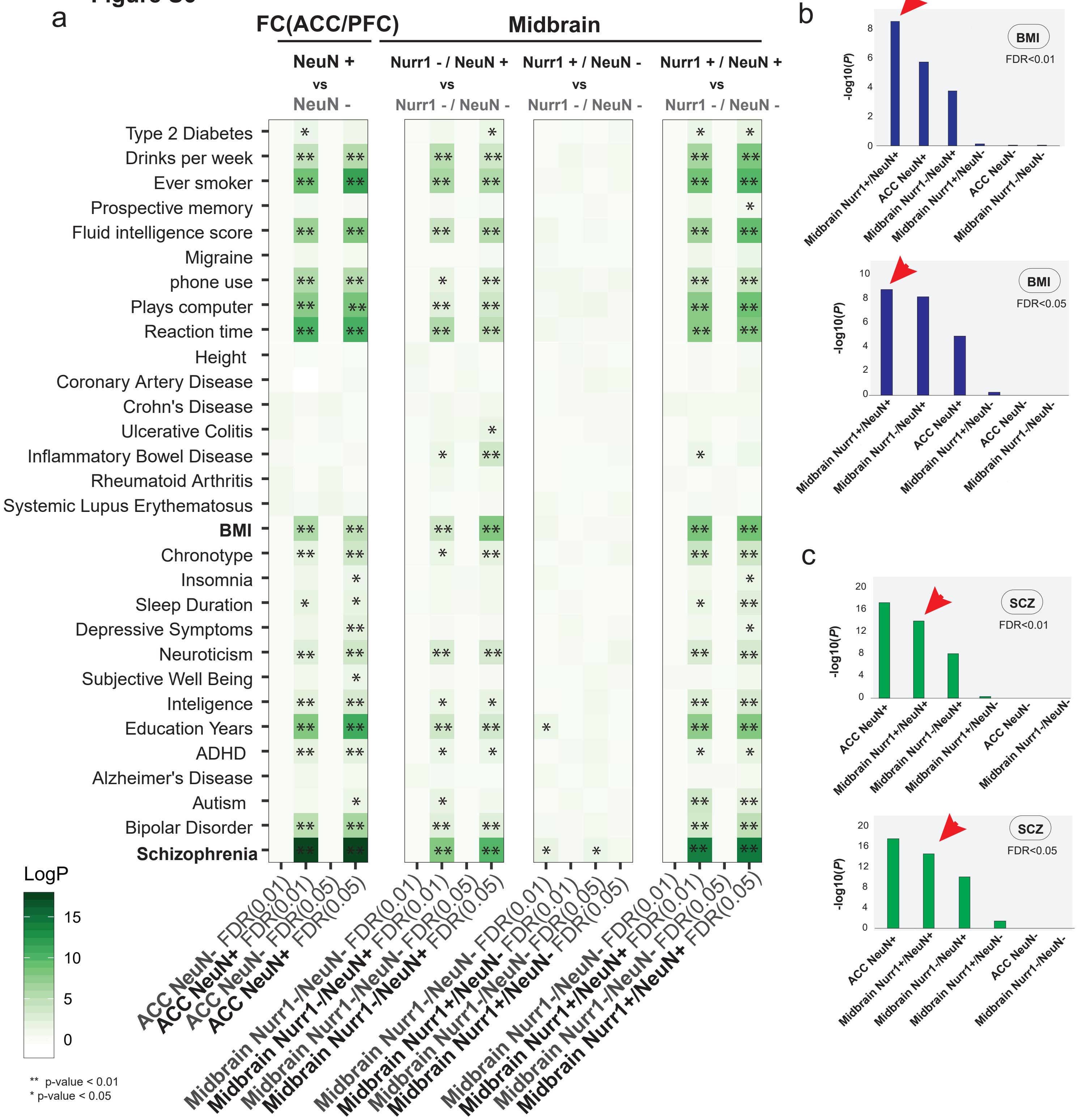
a
FC(ACC/PFC)
Midbrain
NeuN + vs NeuN -
Nurr1 - / NeuN + vs Nurr1 - / NeuN -
Nurr1 + / NeuN - vs Nurr1 - / NeuN -
Nurr1 + / NeuN + vs Nurr1 - / NeuN -
Type 2 Diabetes
Drinks per week
Ever smoker
Prospective memory
Fluid intelligence score
Migraine
phone use
Plays computer
Reaction time
Height
Coronary Artery Disease
Crohn's Disease
Ulcerative Colitis
Inflammatory Bowel Disease
Rheumatoid Arthritis
Systemic Lupus Erythematosus
BMI
Chronotype
Insomnia
Sleep Duration
Depressive Symptoms
Neuroticism
Subjective Well Being
Inteligence
Education Years
ADHD
Alzheimer's Disease
Autism
Bipolar Disorder
Schizophrenia
ACC NeuN- FDR(0.01)
ACC NeuN+ FDR(0.01)
ACC NeuN- FDR(0.05)
ACC NeuN+ FDR(0.05)
Midbrain Nurr1-/NeuN- FDR(0.01)
Midbrain Nurr1-/NeuN+ FDR(0.01)
Midbrain Nurr1-/NeuN- FDR(0.05)
Midbrain Nurr1-/NeuN+ FDR(0.05)
Midbrain Nurr1-/NeuN- FDR(0.01)
Midbrain Nurr1+/NeuN- FDR(0.01)
Midbrain Nurr1-/NeuN- FDR(0.05)
Midbrain Nurr1+/NeuN- FDR(0.05)
Midbrain Nurr1-/NeuN- FDR(0.01)
Midbrain Nurr1+/NeuN+ FDR(0.01)
Midbrain Nurr1-/NeuN- FDR(0.05)
Midbrain Nurr1+/NeuN+ FDR(0.05)
LogP
15
10
5
0
** p-value < 0.01
* p-value < 0.05
b
BMI
FDR<0.01
-log10(P)
Midbrain Nurr1+/NeuN+
ACC NeuN+
Midbrain Nurr1-/NeuN+
Midbrain Nurr1+/NeuN-
ACC NeuN-
Midbrain Nurr1-/NeuN-
BMI
FDR<0.05
-log10(P)
Midbrain Nurr1+/NeuN+
Midbrain Nurr1-/NeuN+
ACC NeuN+
Midbrain Nurr1+/NeuN-
ACC NeuN-
Midbrain Nurr1-/NeuN-
c
SCZ
FDR<0.01
-log10(P)
ACC NeuN+
Midbrain Nurr1+/NeuN+
Midbrain Nurr1-/NeuN+
Midbrain Nurr1+/NeuN-
ACC NeuN-
Midbrain Nurr1-/NeuN-
SCZ
FDR<0.05
-log10(P)
ACC NeuN+
Midbrain Nurr1+/NeuN+
Midbrain Nurr1-/NeuN+
Midbrain Nurr1+/NeuN-
ACC NeuN-
Midbrain Nurr1-/NeuN-

***Supplementary Figure 6. Trait association of expressed genes in six neuronal and non-neuronal populations collected from midbrain and cerebral cortex*.**

MAGMA GWAS association of expressed genes in neuronal versus non-neuronal populations**. a)** At different FDR thresholds (0.01 and 0.05), genes enriched in each sorted population after differential expression analysis (using edgeR[110] wrapper tool RUVseq[40]) were piped into MAGMA association trait analysis. Color scale represents the log p-value and boxes showing "**": represent p-values significant at 0.01 level after FDR correction of multiple testing across all traits and gene sets; or boxes with " * ": p-values significant at 0.05 level. **b**) BMI and **c**) SCZ associate on ranking for each cell population at selected genes at FDR<0.01 (upper bar plot) or FDR<0.05 (lower barplot). Notice Nurr1^{+}/NeuN^{+} MDN (marked by red arrowhead) rank top among for BMI and second for SCZ, among the six populations of nuclei. The following GWAS summary statistics were used in this analyses (PMID numbers): Type 2 Diabetes (30297969); Drinks per week and Ever smoker(30643258); Prospective memory, Fluid intelligence score, Migraine, Phone use, Gaming defined as Plays computer (31427789); Reaction time (29844566); Height (25282103); Coronary artery disease (26343387); Crohn's disease, Ulcerative Colitis; Inflammatory Bowel disease (26192919); Rheumatoid Arthritis (24390342); Systemic Lupus Erythematosus (26502338); BMI (25673413); Chronotype and Sleep duration (27494321); Insomnia (28604731); Depressive Symptoms, Neuroticism, Subjective Well Being (27089181); Intelligence (28530673); Education Years (28197077); Attention Deficit/Hyperactivity Disorder=ADHD (30478444); Alzheimer's Disease (24162737); Autism (30804558); Depression (); Bipolar Disorder (31043756); SCZ (25056061).

Figure S7

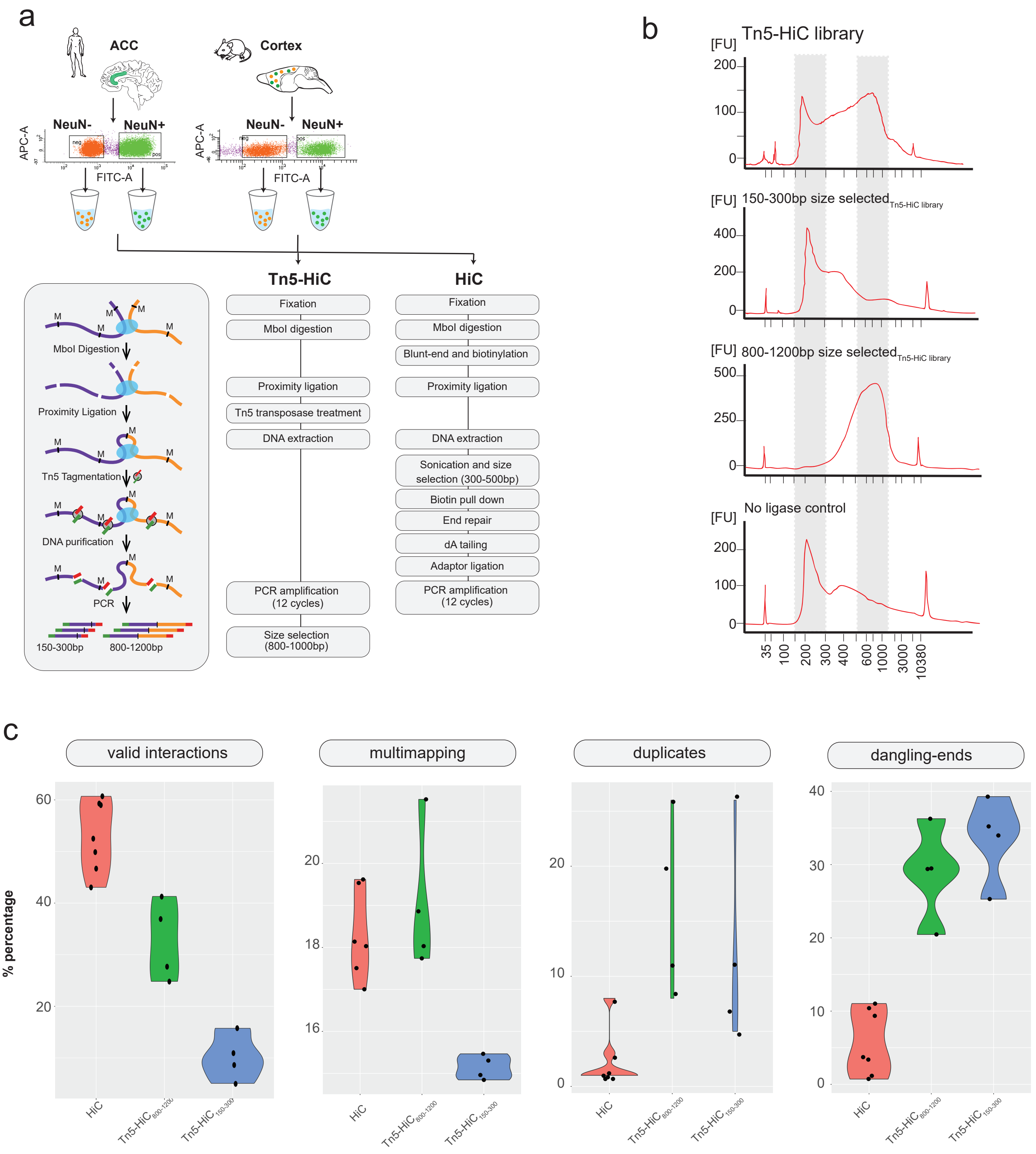
a
ACC
Cortex
NeuN-
NeuN+
APC-A
FITC-A
Tn5-HiC
HiC
Fixation
MboI digestion
Proximity ligation
Tn5 transposase treatment
DNA extraction
PCR amplification (12 cycles)
Size selection (800-1000bp)
Fixation
MboI digestion
Blunt-end and biotinylation
Proximity ligation
DNA extraction
Sonication and size selection (300-500bp)
Biotin pull down
End repair
dA tailing
Adaptor ligation
PCR amplification (12 cycles)
MboI Digestion
Proximity Ligation
Tn5 Tagmentation
DNA purification
PCR
150-300bp
800-1200bp
b
Tn5-HiC library
150-300bp size selected Tn5-HiC library
800-1200bp size selected Tn5-HiC library
No ligase control
[FU]
35
100
200
300
400
600
1000
3000
10380
c
valid interactions
multimapping
duplicates
dangling-ends
% percentage
HiC
Tn5-HiC 800-1200
Tn5-HiC 150-300

***Supplementary Figure 7. ^{Tn5}HiC versus HiC workflow and quality controls*.**

a) ^{Tn5}HiC and HiC procedures for side- by-side comparison of both techniques. Two ^{Tn5}HiC libraries sizes were produced for each cell-type and species with different fragment sizes: 150-300bp and 800-1200bp**.**

b) Bioanalyzer plots of the different fragment libraries ranges produced by ^{Tn5}HiC protocol, from top to bottom plot: without size selection, 150-300bp, 800-1200bp and no ligase control. **c)** Valid interaction and three common artifacts (multi-mapping, dangling ends, duplicates) found in ^{Tn5}HiC method compared to HiC (see also *Additional File 2: Table S1*).

Figure S8

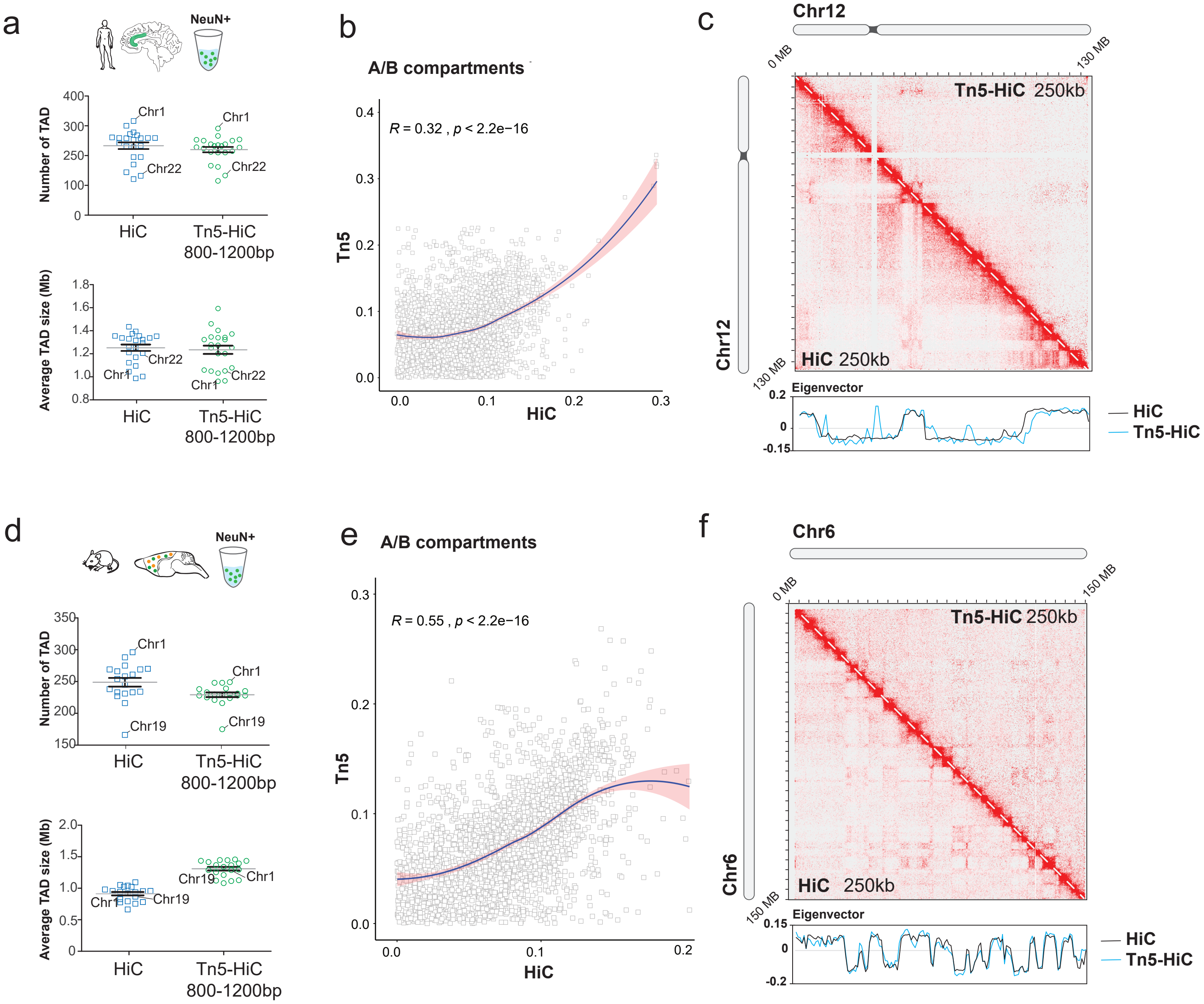
a
NeuN+
Number of TAD
Chr1
Chr22
HiC
Tn5-HiC
800-1200bp
Average TAD size (Mb)
b
A/B compartments
R = 0.32 , p < 2.2e−16
Tn5
HiC
c
Chr12
0 MB
130 MB
Tn5-HiC 250kb
HiC 250kb
Eigenvector
HiC
Tn5-HiC
d
NeuN+
Chr19
e
A/B compartments
R = 0.55 , p < 2.2e−16
f
Chr6
0 MB
150 MB
Tn5-HiC 250kb
HiC 250kb
Eigenvector

Supplementary Figure 8. Topological Associated Domains (TADs) and A/B compartment calling in ^{Tn}5Hi-C and HiC.

Human and mouse NeuN^{+} ^{Tn5}HiC $_{800\text{-}1200bp}$ and conventional HiC libraries were subsampled and bootstrapped to allow for direct comparison. **a, d)** Number of TADs and average size of (top) human and (bottom) mouse libraries, analyzed with TADtree[111]. Each datapoint = 1 autosomal chromosome . **b, e)** Loess regression curve to estimate correlation among A/B compartment calls from HiC and ^{Tn5}HiC libraries, represented by the Pearson correlation values produced by chromosome-wide Eigenvector. **c, f)** Interaction matrix heatmap of human and mouse ^{Tn5}HiC (upper right from diagonal) and HiC (lower left from diagonal) at 250kb resolution. Eigenvector plots showing A/B compartment calls along (top) human chromosome 12 and (bottom) mouse chromosome 6 (blue: ^{Tn5}HiC, black: HiC).

Figure S9

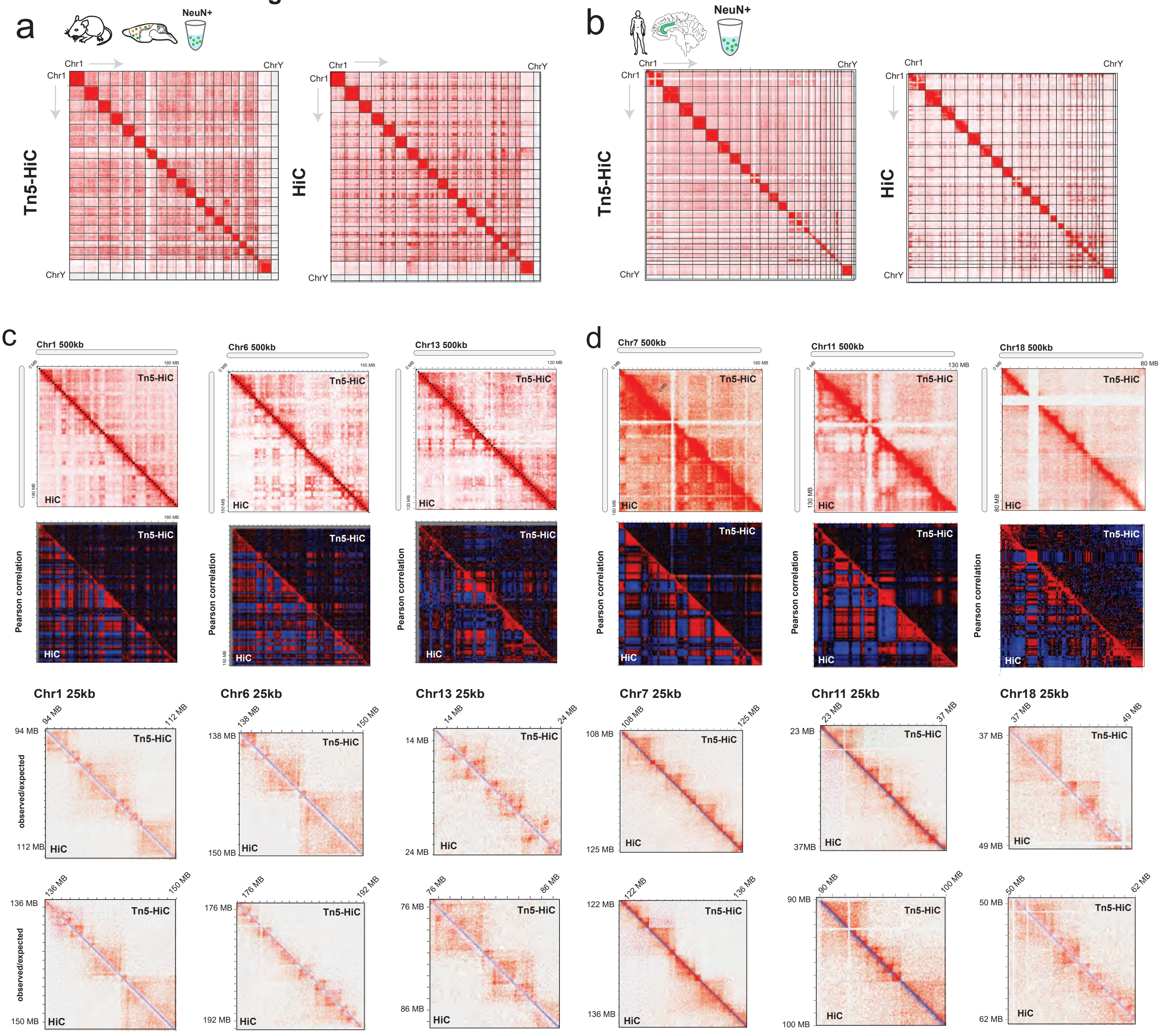
a
NeuN+
Chr1
ChrY
Tn5-HiC
HiC
b
NeuN+
c
Chr1 500kb
Chr6 500kb
Chr13 500kb
Pearson correlation
d
Chr7 500kb
Chr11 500kb
Chr18 500kb
Chr1 25kb
Chr6 25kb
Chr13 25kb
Chr7 25kb
Chr11 25kb
Chr18 25kb
observed/expected
94 MB
112 MB
138 MB
150 MB
14 MB
24 MB
108 MB
125 MB
23 MB
37MB
37 MB
49 MB
136 MB
176 MB
192 MB
76 MB
86 MB
122 MB
90 MB
100 MB
50 MB
62 MB

***Supplementary Figure 9. Neuronal mouse and human Tn5-HiC interactions matrices*.** Interaction matrices examples of subsampled ^{Tn5}HiC and HiC libraries NeuN^{+} samples. **a,b)** Genome-wide mouse (a) and human (b) heatmap interaction matrix at 2.5MB resolution of ^{Tn5}HiC (left) and HiC (right). **c, d)** Interaction matrices of ^{Tn5}HiC and HiC at (top) 500kb and (bottom) 25kb. Upper right from diagonal, ^{Tn5}HiC and lower left from diagonal, HiC library. Also shown are Pearson correlation at 500kb.

Figure S10

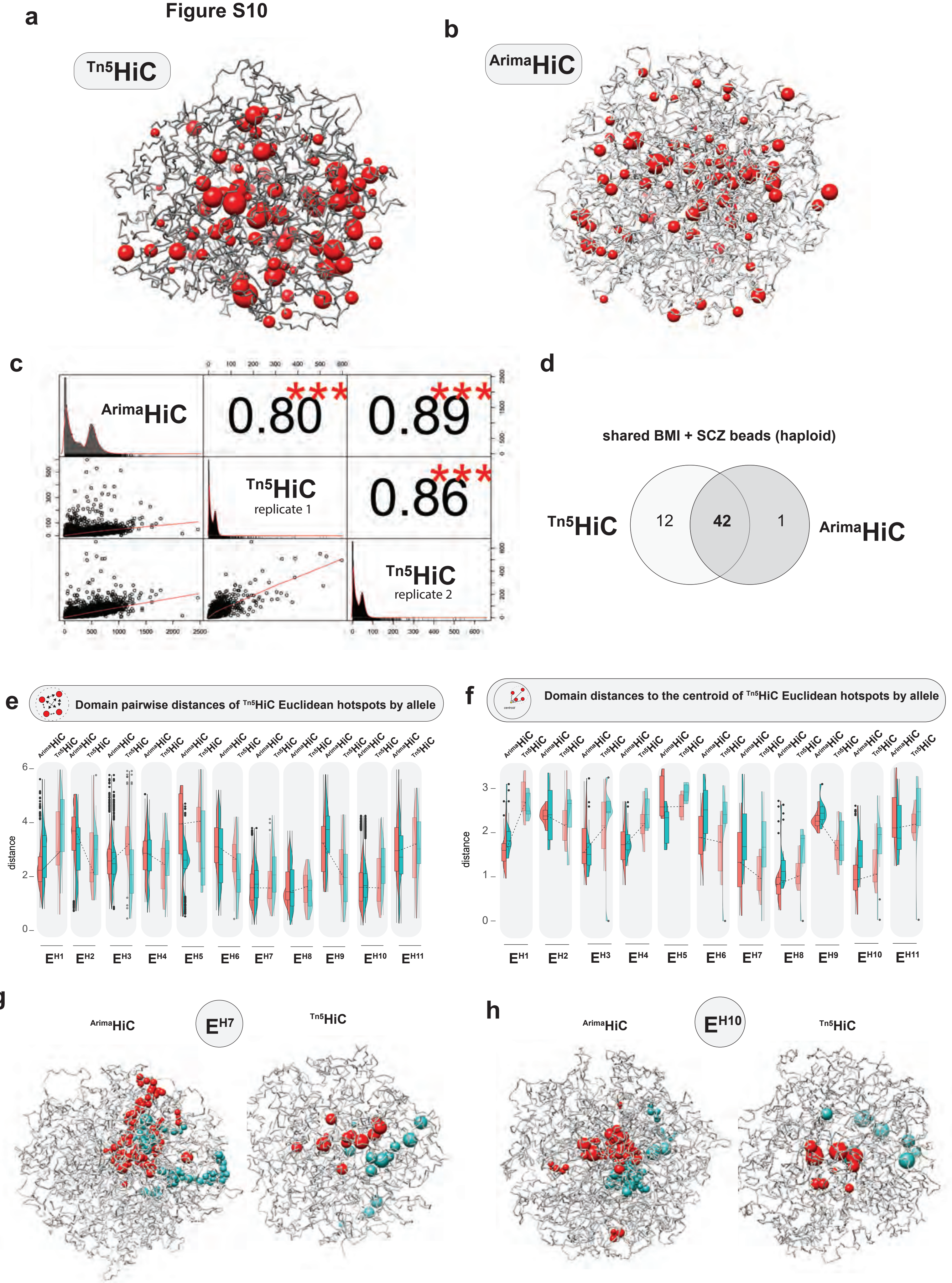

a
Tn5HiC
b
ArimaHiC
c
ArimaHiC
0.80
0.89
Tn5HiC
replicate 1
0.86
Tn5HiC
replicate 2
d
shared BMI + SCZ beads (haploid)
Tn5HiC
12
42
1
ArimaHiC
e
Domain pairwise distances of Tn5HiC Euclidean hotspots by allele
distance
EH1
EH2
EH3
EH4
EH5
EH6
EH7
EH8
EH9
EH10
EH11
f
Domain distances to the centroid of Tn5HiC Euclidean hotspots by allele
distance
EH1
EH2
EH3
EH4
EH5
EH6
EH7
EH8
EH9
EH10
EH11
g
ArimaHiC
EH7
Tn5HiC
h
ArimaHiC
EH10
Tn5HiC

Supplementary Figure 10. Nurr1^{+}/NeuN^{+} ^{Tn5}HiC and ^{Arima}HiC.

Comparison of ^{Tn5}HiC Nurr1^{+}/NeuN^{+} and ^{Arima}HiC Nurr1^{+}/NeuN^{+} domain-based chrom3D models. Note that due the higher proportion of valid interactions pairs (~17% of the total number of pairs of ^{Tn5}HiC versus the ~35% of ^{Arima}HiC libraries, the resolution of the 3D model is considerably higher in ^{Arima}HiC libraries. **a, b)** *In silico* 3D models of MDN Nurr1^{+}/NeuN^{+} spatial genomes. Note that domains (beads) are smaller in ^{Arima}HiC model (**b**, left) compared to ^{Tn5}HiC (**a**, right), reflecting better signal to noise. Average domain width, ^{Arima}Hi-C 1.16Mb ± 1.75 (median=0.9Mb) and ^{Tn5}Hi-C was 1.68MB ± 2.98Mb (median=0.95Mb) **c)** Correlation of intrachromosomal interactions at 40kb resolution genome-wide, of ^{Arima}HiC and two replicates of ^{Tn5}HiC libraries.*** P<0.001. **d)** Venn plot of BMI and schizophrenia domains intersections, showing that 42/43 domains (called by the Arrowhead program in Nurr1^{+}/NeuN^{+} ^{Arima}Hi-C) showing co-localization of BMI and SCZ risk sequences fully overlap with the 50 shared domains in Nurr1^{+}/NeuN^{+} ^{Tn5}Hi-C. **e-g)** chrom3D diploid models of MDN Nurr1^{+}/NeuN^{+} ^{Arima}Hi-C and ^{Tn5}Hi-C. Alleles represented by red and green colors, respectively. **e, f**) ^{Arima}HiC and ^{Tn5}HiC Euclidean distance comparison of **e**) pair-wise distances among distinct domains comprising Euclidean hotspots E^{H1} – E^{H11} of the ^{Tn5}HiC Nurr1^{+}/NeuN^{+} model and **f**) distances to the centroid for each hotspot (E^{H1} – E^{H11}) domains. **g)** Euclidean hotspot E^{H7} and **h)** Euclidean hotspot E^{H10} mapped in (left) ^{Arima}HiC and (right) ^{Tn5}Hi-C chrom3D diploid models.

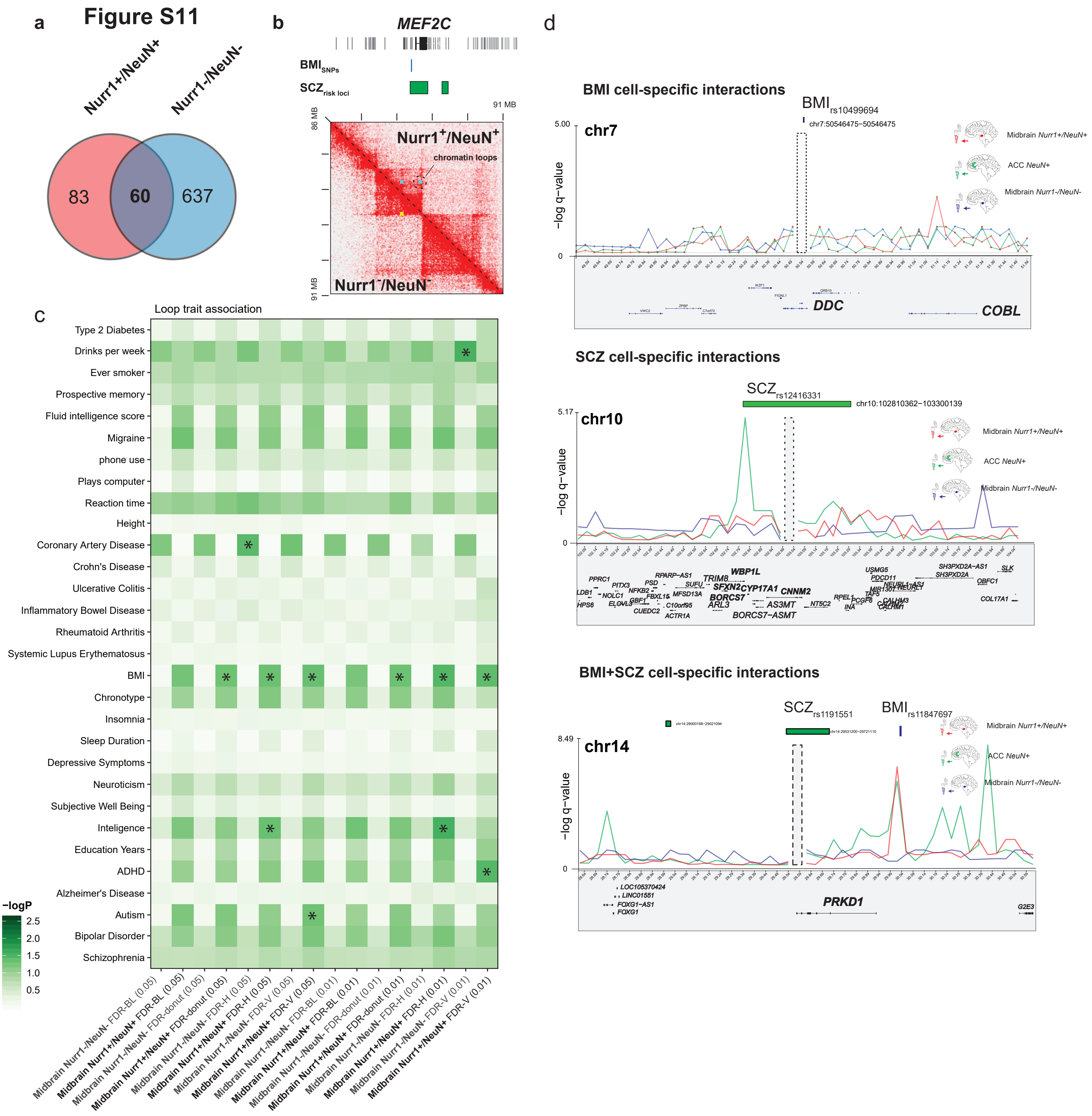
a
Figure S11
Nurr1+/NeuN+
Nurr1-/NeuN-
83
60
637
b
MEF2C
BMI SNPs
SCZ risk loci
91 MB
86 MB
Nurr1+/NeuN+
chromatin loops
Nurr1-/NeuN-
C
Loop trait association
Type 2 Diabetes
Drinks per week
Ever smoker
Prospective memory
Fluid intelligence score
Migraine
phone use
Plays computer
Reaction time
Height
Coronary Artery Disease
Crohn's Disease
Ulcerative Colitis
Inflammatory Bowel Disease
Rheumatoid Arthritis
Systemic Lupus Erythematosus
BMI
Chronotype
Insomnia
Sleep Duration
Depressive Symptoms
Neuroticism
Subjective Well Being
Inteligence
Education Years
ADHD
Alzheimer's Disease
Autism
Bipolar Disorder
Schizophrenia
-logP
2.5
2.0
1.5
1.0
0.5
Midbrain Nurr1-/NeuN- FDR-BL (0.05)
Midbrain Nurr1+/NeuN+ FDR-BL (0.05)
Midbrain Nurr1-/NeuN- FDR-donut (0.05)
Midbrain Nurr1+/NeuN+ FDR-donut (0.05)
Midbrain Nurr1-/NeuN- FDR-H (0.05)
Midbrain Nurr1+/NeuN+ FDR-H (0.05)
Midbrain Nurr1-/NeuN- FDR-V (0.05)
Midbrain Nurr1+/NeuN+ FDR-V (0.05)
Midbrain Nurr1-/NeuN- FDR-BL (0.01)
Midbrain Nurr1+/NeuN+ FDR-BL (0.01)
Midbrain Nurr1-/NeuN- FDR-donut (0.01)
Midbrain Nurr1+/NeuN+ FDR-donut (0.01)
Midbrain Nurr1-/NeuN- FDR-H (0.01)
Midbrain Nurr1+/NeuN+ FDR-H (0.01)
Midbrain Nurr1-/NeuN- FDR-V (0.01)
Midbrain Nurr1+/NeuN+ FDR-V (0.01)
d
BMI cell-specific interactions
BMI rs10499694
chr7:50546475-50546475
5.00
chr7
-log q-value
0
Midbrain Nurr1+/NeuN+
ACC NeuN+
Midbrain Nurr1-/NeuN-
DDC
COBL
SCZ cell-specific interactions
SCZ rs12416331
chr10:102810362-103300139
5.17
chr10
-log q-value
0
Midbrain Nurr1+/NeuN+
ACC NeuN+
Midbrain Nurr1-/NeuN-
BMI+SCZ cell-specific interactions
SCZ rs1191551
BMI rs11847697
8.49
chr14
-log q-value
0
Midbrain Nurr1+/NeuN+
ACC NeuN+
Midbrain Nurr1-/NeuN-
PRKD1

Supplementary Figure 11. Cell-specific chromosomal conformations enriched for cognitive and psychiatric traits

a) Venn intersection among Nurr1$^+$/NeuN$^+$ and Nurr1$^-$/NeuN$^-$ HICCUPS[54] for loops called significant both at 5 and 10kb resolution. **b**) Example of loop called for *MEF2C* genomic locus associated with BMI and schizophrenia risk. Above diagonal heatmap (25kb resolution) shows midbrain Nurr1$^+$/NeuN$^+$ contact map with two chromatin loops (marked in blue) and below diagonal heatmap shows Nurr1$^-$/NeuN$^-$ single loop. **c**) LD-score partitioned heritability[61] plot using for midbrain Nurr1$^+$/NeuN$^+$ and Nurr1$^-$/NeuN$^-$ loops at 0.01 and 0.05 FDR thresholds (HICCUPS[54]). **d**) Binomial chromosomal contact mapping at the site of specific BMI and SCZ risk polymorphisms. Contact enrichment over the genomic region of the highlighted bin (vertical gray bar) as indicated. Lines represent contact enrichments, (red) midbrain Nurr1$^+$/NeuN$^+$, (green) FC/ACC NeuN$^+$ and (blue) midbrain Nurr1$^-$/NeuN$^-$.

Figure S12

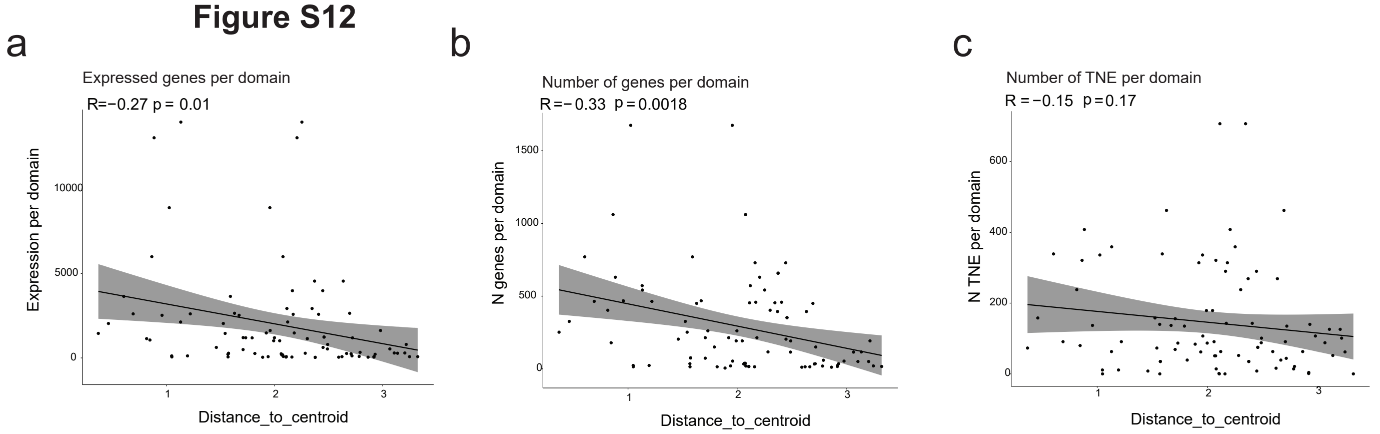

a
Expressed genes per domain
R=−0.27 p = 0.01
Expression per domain
10000
5000
0
1
2
3
Distance_to_centroid
b
Number of genes per domain
R =− 0.33 p = 0.0018
N genes per domain
1500
1000
500
0
1
2
3
Distance_to_centroid
c
Number of TNE per domain
R = −0.15 p = 0.17
N TNE per domain
600
400
200
0
1
2
3
Distance_to_centroid

Supplementary Figure 12. Gene expression to nuclear centroid correlation analysis.

Correlations plots for gene expression, number of genes and TNE transcribed non-coding element per domain in relation with the proximity to the nuclear centroid. **a**) Expressed genes per domain to nuclear centroid. **b**) Number of genes per domain to nuclear centroid. **c**) Number of TNE per domain to nuclear centroid. For all plots, the 100 domains carrying both BMI and SCZ risk SNPs were used as input.

Figure S13

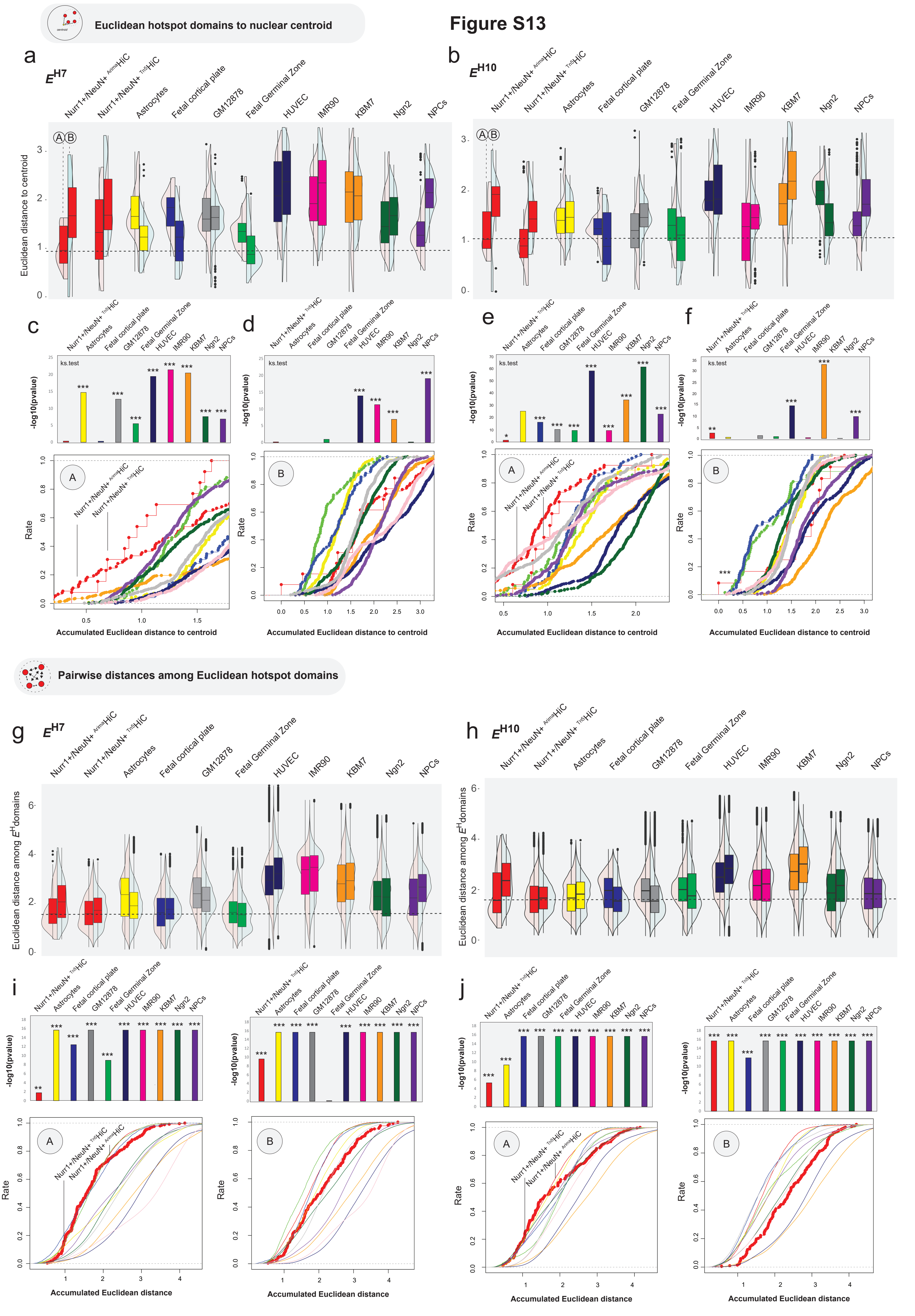
Euclidean hotspot domains to nuclear centroid
centroid
a
b
E^{H7}
E^{H10}
Nurr1+/NeuN+ ArimaHiC
Nurr1+/NeuN+ Tn5HiC
Astrocytes
Fetal cortical plate
GM12878
Fetal Germinal Zone
HUVEC
IMR90
KBM7
Ngn2
NPCs
A
B
Euclidean distance to centroid
c
d
e
f
ks.test
-log10(pvalue)
Rate
Accumulated Euclidean distance to centroid
Pairwise distances among Euclidean hotspot domains
g
h
Euclidean distance among E^{H}domains
i
j
Accumulated Euclidean distance

Supplementary Figure 13. Cell-type specific conformations of Euclidean hotspots E^{H7} and E^{H10} across multiple cell types.

(a-f) Nuclear centroid and **(g-j)** pairwise distances of domains comprising E^{H7} and E^{H10} across 11 HiC datasets including two types of MDN Nurr1^{+}/NeuN^{+} libraries (^{Arima}Hi-C and ^{Tn5}Hi-C) , and 9 previously published Hi-C datasets generated from various other cell lines and tissues. (**a,g)** E^{H7} and **b,h**) E^{H10} violin plots of (**a,b**) distance-to- nuclear centroid and (**g,h**) pairwise distances among hotspot domains in chrom3D diploid models, shown for both alleles 'A' and 'B' across multiple cell types, from left to right: (red boxplots) MDN Nurr1^{+}/NeuN^{+} ^{Arima}HiC , MDN Nurr1^{+}/NeuN^{+} ^{Tn5}HiC; (yellow boxplots) stem cell-derived astrocytes[28]; (navy blue boxplots) fetal brain cortical plate[24]; (gray boxplots) GM12878 cell line[30]; (green boxplots) fetal brain germinal zone[24]; (dark blue, pink and orange boxplots) HUVEC, IMR90 and KBM7 cell lines[30]; (green and purple boxplots) Neurogenin 2 (NGN2) induced neurons and neural precursor cells (NPC)[28]. Dashed line denotes median of 'A' domain distance-to-centroid for ^{Arima}HiC Nurr1^{+}/NeuN^{+}. Note that E^{H7} is biased towards A alleles while E^{H10} is 'monoallelic'. **c, d)** E^{H7} and **e,f**) E^{H10} ^{Arima}Hi-C library-to-other library comparison of domain distances to centroid shown separately for **c,e)** 'A' and **d,f**) 'B' alleles. **i)** E^{H7} and **j**) E^{H10} ^{Arima}Hi-C library-to-other library comparison of pairwise distances among hotspot domains shown separately (left) 'A' and (right) 'B' alleles. **c-f**) and **i,j**) (Kolmogorov–Smirnov test, *(**, ***) P< 0.05(0.01, 0.001).

Figure S14

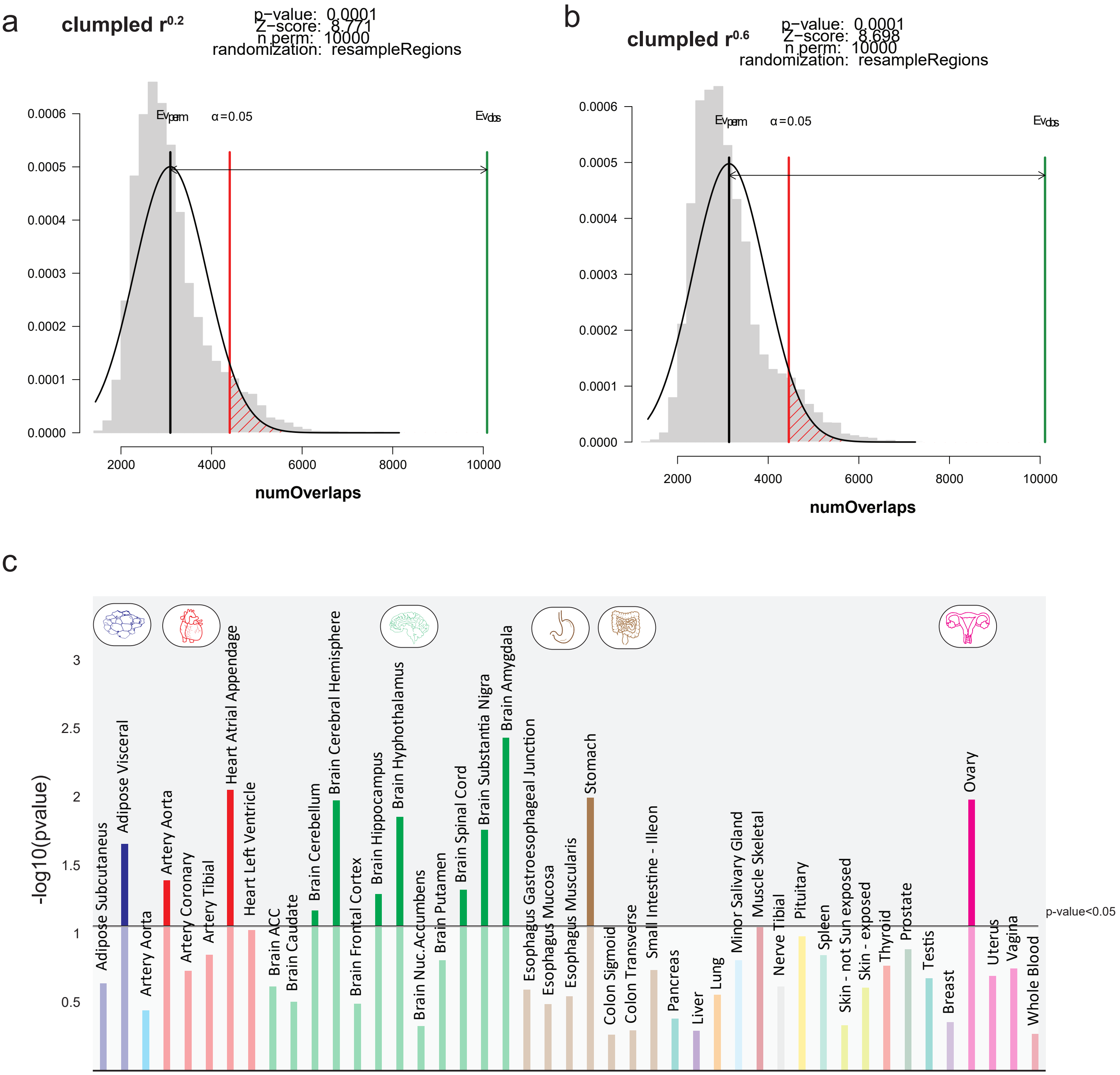
a
clumpled $r^{0.2}$
p−value: 0.0001
Z−score: 8.771
n perm: 10000
randomization: resampleRegions
Evperm
α=0.05
Evobs
numOverlaps
b
clumpled $r^{0.6}$
p−value: 0.0001
Z−score: 8.698
n perm: 10000
randomization: resampleRegions
Evperm
α=0.05
Evobs
numOverlaps
c
-log10(pvalue)
p-value<0.05
Adipose Subcutaneus
Adipose Visceral
Artery Aorta
Artery Aorta
Artery Coronary
Artery Tibial
Heart Atrial Appendage
Heart Left Ventricle
Brain ACC
Brain Caudate
Brain Cerebellum
Brain Cerebral Hemisphere
Brain Frontal Cortex
Brain Hippocampus
Brain Hyphothalamus
Brain Nuc.Accumbens
Brain Putamen
Brain Spinal Cord
Brain Substantia Nigra
Brain Amygdala
Esophagus Gastroesophageal Junction
Esophagus Mucosa
Esophagus Muscularis
Stomach
Colon Sigmoid
Colon Transverse
Small Intestine - Illeon
Pancreas
Liver
Lung
Minor Salivary Gland
Muscle Skeletal
Nerve Tibial
Pituitary
Spleen
Skin - not Sun exposed
Skin - exposed
Thyroid
Prostate
Testis
Breast
Ovary
Uterus
Vagina
Whole Blood

Supplementary Figure 14: '**Cross-disorder' chromosomal contacts interconnecting BMI with SCZ risk variants are enriched for tissue-specific eQTLs.**

a,b) eQTL from EH 1-11, with clumping for each gene using the PLINK 1.90 software at a clump distance of 250kb and an r^2 of **a**) 0.2 and **b**) 0.6. Notice significant enrichment for eQTLs at the E^H 1-11. **c)** BMI-SCZ contacts associated with GTEX cis eQTLs unique to specific tissues, as indicated. Note significant enrichment for brain and adipocyte-rich tissues.
